# Supplementary material for: Protocol for a systematic review on routine use of antibiotics for infants less than 6 months of age with growth failure/faltering
Source: BMJ Open. 2022 Jun 30;12(6):e057241. doi: 10.1136/bmjopen-2021-057241 (PMC9247651; doi:10.1136/bmjopen-2021-057241)
Supplement: Supplementary data [file bmjopen-2021-057241supp001.pdf]

Appendix 1

Search Strategies for Growth Failure AND Antibiotics

PubMed

(("Infant"[MeSH Terms] OR "Child"[MeSH Terms:noexp] OR "Premature
Birth"[MeSH Terms] OR
("infant*"[Title/Abstract] OR "infancy"[Title/Abstract] OR
"baby"[Title/Abstract] OR
"babies"[Title/Abstract] OR "neonat*"[Title/Abstract] OR "neo
nat*"[Title/Abstract] OR
"newborn*"[Title/Abstract] OR "new born*"[Title/Abstract] OR "newly
born*"[Title/Abstract] OR
"preterm"[Title/Abstract] OR "preterms"[Title/Abstract] OR "pre
term"[Title/Abstract] OR "pre
terms"[Title/Abstract] OR "lbw"[Title/Abstract] OR "vlbw"[Title/Abstract] OR
"elbw"[Title/Abstract]
OR "child*"[Title/Abstract] OR "youth"[Title/Abstract] OR
"juvenile*"[Title/Abstract]) OR
("premature*"[Title/Abstract] AND ("birth*"[Title/Abstract] OR
"born"[Title/Abstract] OR
"deliver*"[Title/Abstract])) OR ("low"[Title/Abstract] AND
("birthweight*"[Title/Abstract] OR "birth
weight*"[Title/Abstract]))) AND ("Malnutrition"[MeSH Terms] OR "Failure to
Thrive"[MeSH Terms]
OR "Growth Disorders"[MeSH Terms:noexp] OR "Severe Acute Malnutrition"[MeSH
Terms] OR
"Wasting Syndrome"[MeSH Terms:noexp] OR "Infant Nutrition Disorders"[MeSH
Terms:noexp]
OR ("growth failure*"[Title/Abstract] OR "growth falter*"[Title/Abstract] OR "slow
growth"[Title/Abstract] OR "slowed growth"[Title/Abstract] OR
"Malnutrition"[Title/Abstract] OR
"malnourish*"[Title/Abstract] OR "Severe Acute Malnutrition"[Title/Abstract] OR
"SAM"[Title/Abstract] OR "wasting"[Title/Abstract] OR
"wasted"[Title/Abstract] OR "growth
restrict*"[Title/Abstract] OR "growth retard*"[Title/Abstract] OR "Failure to
Thrive"[Title/Abstract]
OR "FTT"[Title/Abstract] OR "growth disorder*"[Title/Abstract] OR "growth
arrest*"[Title/Abstract]
OR "growth deficien*"[Title/Abstract] OR "growth disturb*"[Title/Abstract])) AND
("Anti-Bacterial
Agents"[Pharmacological Action] OR "Anti-Bacterial Agents"[MeSH Terms] OR
("antibiotic*"[Title/Abstract] OR "anti bacterial agent*"[Title/Abstract] OR
"antibacterial*"[Title/Abstract] OR "anti bacterial*"[Title/Abstract] OR
"bactericid*"[Title/Abstract]
OR "bacteriocid*"[Title/Abstract] OR "anti mycobacterial*"[Title/Abstract] OR
"antimycobacterial*"[Title/Abstract]))) NOT ("Animals"[MeSH Terms] NOT
("Animals"[MeSH
Terms] AND "Humans"[MeSH Terms]))

CINAHL

S1 (MH "Infant+")
S2 (MH "Child")
S3 (MH "Childbirth, Premature")
S4 TI (infant* OR infancy OR baby OR babies OR neonat* OR "neo nat*" OR newborn* OR "new

born*" OR "newly born*" OR preterm OR preterms OR "pre term" OR "pre terms" OR lbw OR vlbw
OR elbw OR child* OR youth OR juvenile*)
S5 AB (infant* OR infancy OR baby OR babies OR neonat* OR "neo nat*" OR newborn* OR "new born*" OR "newly born*" OR preterm OR preterms OR "pre term" OR "pre terms" OR lbw OR vlbw
OR elbw OR child* OR youth OR juvenile*)
S6 TI (premature* N2 (birth* OR born OR deliver*))
S7 AB (premature* N2 (birth* OR born OR deliver*))
S8 TI (low N2 (birthweight* OR "birth weight*"))
S9 AB (low N2 (birthweight* OR "birth weight*"))
S10 S1 OR S2 OR S3 OR S4 OR S5 OR S6 OR S7 OR S8 OR S9

S11 (MH "Malnutrition")
S12 (MH "Failure to Thrive")
S13 (MH "Growth Disorders")
S14 (MH "Wasting Syndrome")
S15 (MH “Infant Nutrition Disorders”)
S16 TI ((grow*) N2 (fail* OR falter* OR slow* OR restrict* OR retard* OR deficien* OR arrest* OR disturb* OR disorder*))
S17 AB ((grow*) N2 (fail* OR falter* OR slow* OR restrict* OR retard* OR deficien* OR arrest* OR disturb* OR disorder*))
S18 TI ((fail*) W2 (thrive))
S19 AB ((fail*) W2 (thrive))
S20 TI (Malnutrition OR malnourish* OR "Severe Acute Malnutrition" OR SAM OR wasting OR wasted OR FTT)
S21 AB (Malnutrition OR malnourish* OR "Severe Acute Malnutrition" OR SAM OR wasting OR wasted OR FTT)
S22 S11 OR S12 OR S13 OR S14 OR S15 OR S16 OR S17 OR S18 OR S19 OR S20 OR S21
S23 (MH "Antibiotics+")
S24 TI (antibiotic* OR "anti bacterial*" OR "antibacterial*" OR bactericid*OR bacteriocid* OR "anti
mycobacterial*" OR "antimycobacterial*")
S25 AB (antibiotic* OR "anti bacterial*" OR "antibacterial*" OR bactericid* OR bacteriocid* OR
"anti mycobacterial*" OR "antimycobacterial*")
S26 S23 OR S24 OR S25
S27 (MH "Animals") NOT ((MH "Animals") AND (MH "Human"))
S28 S10 AND S22 AND S26
S29 S28 NOT S27
S30 S28 NOT S27 Exclude Medline Records
Embase
#1'infant'/exp OR 'newborn'/exp OR 'prematurity'/exp OR 'low birth weight'/exp OR 'very low birth
weight'/exp OR 'extremely low birth weight'/exp OR 'juvenile'/de OR 'child'/de
#2 infant*:ti,ab OR infancy:ti,ab OR baby:ti,ab OR babies:ti,ab OR neonat*:ti,ab OR 'neo nat*':ti,ab OR newborn*:ti,ab OR 'new born*':ti,ab OR 'newly born*':ti,ab OR preterm:ti,ab OR preterms:ti,ab
OR 'pre term':ti,ab OR 'pre terms':ti,ab OR lbw:ti,ab OR vlbw:ti,ab OR elbw:ti,ab OR child*:ti,ab OR
youth:ti,ab OR juvenile*:ti,ab
#3 (premature* NEAR/2 (birth* OR born OR deliver*)):ti,ab

BMJ Publishing Group Limited (BMJ) disclaims all liability and responsibility arising from any reliance placed on this supplemental material which has been supplied by the author(s)

#4 (low NEAR/2 (birthweight* OR 'birth weight*')):ti,ab
#5 #1 OR #2 OR #3 OR #4

#6 'malnutrition'/exp OR 'failure to thrive'/exp OR 'growth disorder'/de OR 'wasting syndrome'/de
OR 'growth retardation'/de
#7(grow* NEAR/2 (fail* OR falter* OR slow* OR restrict* OR retard* OR deficien* OR arrest* OR disturb* OR disorder*)):ti,ab
#8 (fail* NEXT/2 thrive):ti,ab
#9 malnutrition:ti,ab OR malnourish*:ti,ab OR 'severe acute malnutrition':ti,ab OR sam:ti,ab OR wasting:ti,ab OR wasted:ti,ab OR ftt:ti,ab
#10 #6 OR #7 OR #8 OR #9
#11'antibiotic agent'/exp OR 'bactericide'/de OR 'antimycobacterial agent'/de
#12 antibiotic*:ti,ab OR 'anti bacterial*':ti,ab OR 'antibacterial*':ti,ab OR bactericid*:ti,ab OR bacteriocid*:ti,ab OR antimycobacterial*:ti,ab OR 'anti mycobacterial*':ti,ab
#13 #11 OR #12
#14 #5 AND #10 AND #13
#15 #14 NOT ('animals'/exp NOT 'humans'/exp)
#16 #15 NOT [medline]/lim

CENTRAL

#1 MeSH descriptor: [Infant] explode all trees
#2 MeSH descriptor: [Child] this term only
#3 MeSH descriptor: [Premature Birth] explode all trees
#4 infant*:ti,ab OR infancy:ti,ab OR baby:ti,ab OR babies:ti,ab OR neonat*:ti,ab OR newborn*:ti,ab OR preterm:ti,ab OR preterms:ti,ab OR lbw:ti,ab OR vlbw:ti,ab OR elbw:ti,ab OR child*:ti,ab OR youth:ti,ab OR juvenile*:ti,ab
#5 (neo NEXT nat* OR new NEXT born* OR newly NEXT born* OR pre NEXT term OR pre NEXT terms):ti,ab
#6 (premature* NEAR/2 (birth* OR born OR deliver*)):ti,ab
#7 (low NEAR/2 (birthweight* OR birth NEXT weight*)):ti,ab
#8 #1 OR #2 OR #3 OR #4 OR #5 OR #6 OR #7
#9 MeSH descriptor: [Malnutrition] explode all trees
#10 MeSH descriptor: [Failure to Thrive] explode all trees
#11 MeSH descriptor: [Growth Disorders] this term only
#12 MeSH descriptor: [Severe Acute Malnutrition] explode all trees
#13 MeSH descriptor: [Wasting Syndrome] this term only
#14 MeSH descriptor: [Infant Nutrition Disorders] this term only
#15 ((grow*) NEAR/2 (fail* OR falter* OR slow* OR restrict* OR retard* OR deficien* OR arrest* OR disturb* OR disorder*)):ti,ab

#16 ((fail*) NEXT/2 (thrive)):ti,ab
#17 (severe NEXT acute NEXT malnutrition):ti,ab
#18 malnutrition:ti,ab OR malnourish*:ti,ab OR sam:ti,ab OR wasting:ti,ab OR wasted:ti,ab OR ftt:ti,ab
#19 #9 OR #10 OR #11 OR #12 OR #13 OR #14 OR #15 OR #16 OR #17 OR #18
#20 MeSH descriptor: [Anti-Bacterial Agents] explode all trees
#21 antibiotic*:ti,ab OR antibacterial*:ti,ab OR bactericid*:ti,ab OR bacteriocid*:ti,ab OR antimycobacterial*:ti,ab
#22 (anti NEXT bacterial*):ti,ab
#23 (anti NEXT mycobacterial*):ti,ab
#24 #20 OR #21 OR #22 OR #23
#25 #8 AND #19 AND #24
#26 MeSH descriptor: [Animals] explode all trees

#27 MeSH descriptor: [Humans] explode all trees
#28 (#26 NOT (#26 AND #27))
#29 #25 NOT #28
#30 "accession number" NEAR pubmed
#31 #29 NOT #30

Scopus

((TITLE-ABS (infant* OR infancy OR baby OR babies OR neonat* OR "neo nat*" OR
newborn* OR "new born*" OR "newly born*" OR preterm OR preterms OR "pre term" OR
"pre terms" OR lbw OR vlbw OR elbw OR child* OR youth OR juvenile*)) OR (TITLE-ABS
(low W/2 (birthweight* OR "birth weight*"))) OR (TITLE-ABS (premature* W/2 (birth* OR
born OR deliver*)))) AND ((TITLE-ABS ((grow*) W/2 (fail* OR falter* OR slow* OR
restrict* OR retard* OR deficien* OR arrest* OR disturb* OR disorder*))) OR (TITLE-ABS (
(fail*) PRE/2 (thrive))) OR (TITLE-ABS (malnutrition OR malnourish* OR "Severe Acute
Malnutrition" OR sam OR wasting OR wasted OR ftt))) AND (TITLE-ABS (antibiotic* OR
"anti bacterial*" OR "antibacterial*" OR bactericid* OR bacteriocid* OR "anti mycobacterial*"
OR "antimycobacterial*")) AND NOT INDEX (medline)

Web of Science

#1 TI=(infant* OR infancy OR baby OR babies OR neonat* OR "neo nat*" OR newborn* OR
"new born*" OR "newly born*" OR preterm OR preterms OR "pre term" OR "pre terms" OR
lbw OR vlbw OR elbw OR child* OR youth OR juvenile*)
#2 AB=(infant* OR infancy OR baby OR babies OR neonat* OR "neo nat*" OR newborn* OR
"new born*" OR "newly born*" OR preterm OR preterms OR "pre term" OR "pre terms" OR
lbw OR vlbw OR elbw OR child* OR youth OR juvenile*)
#3 TI=(premature* NEAR/2 (birth* OR born OR deliver*))

#4 AB=(premature* NEAR/2 (birth* OR born OR deliver*))
#5 TI=(low NEAR/2 (birthweight* OR "birth weight*"))
#6 AB=(low NEAR/2 (birthweight* OR "birth weight*"))
#7 #6 OR #5 OR #4 OR #3 OR #2 OR #1
#8 TI=((grow*) NEAR/2 (fail* OR falter* OR slow* OR restrict* OR retard* OR deficien* OR arrest*
OR disturb* OR disorder*))
#9 AB=((grow*) NEAR/2 (fail* OR falter* OR slow* OR restrict* OR retard* OR deficien* OR arrest*
OR disturb* OR disorder*))
#10 TI=((fail*) NEAR/2 (thrive))
#11 AB=((fail*) NEAR/2 (thrive))
#12 TI =(Malnutrition OR malnourish* OR "Severe Acute Malnutrition" OR SAM OR wasting OR
wasted OR FTT)
#13 AB=(Malnutrition OR malnourish* OR "Severe Acute Malnutrition" OR SAM OR wasting OR
wasted OR FTT)
#14 #13 OR #12 OR #11 OR #10 OR #9 OR #8
#15 TI=(antibiotic* OR "anti bacterial*" OR "antibacterial*" OR bactericid* OR bacteriocid* OR "anti
mycobacterial*" OR "antimycobacterial*")
#16 AB=(antibiotic* OR "anti bacterial*" OR "antibacterial*" OR bactericid* OR bacteriocid* OR
"anti mycobacterial*" OR "antimycobacterial*")
#17 #16 OR #15
#18 #17 AND #14 AND #7

LILACS

(mh:(infant)) OR ((mh:(child))) OR ((mh:("Premature Birth"))) OR ((ti:((premature* AND (birth* OR born OR deliver*))))) OR ((ab:((premature* AND (birth* OR born OR deliver*))))) OR ((ti:((low AND (birthweight* OR "birth weight*"))))) OR ((ab:((low AND (birthweight* OR "birth weight*"))))) OR
((ti:(infant* OR infancy OR baby OR babies OR neonat* OR "neo nat*" OR newborn* OR "new born*" OR "newly born*" OR preterm OR preterms OR "pre term" OR "pre terms" OR lbw OR vlbw
OR elbw OR child* OR youth OR juvenile*))) OR ((ab:(infant* OR infancy OR baby OR babies OR
neonat* OR "neo nat*" OR newborn* OR "new born*" OR "newly born*" OR preterm OR preterms
OR "pre term" OR "pre terms" OR lbw OR vlbw OR elbw OR child* OR youth OR juvenile*)))
AND
((mh:(malnutrition)) OR ((mh:("Failure to Thrive"))) OR ((mh:("Growth Disorders"))) OR ((mh:("Severe Acute Malnutrition"))) OR ((mh:("Wasting Syndrome"))) OR ((mh:("Infant Nutrition
Disorders"))) OR ((ti:("growth failure*" OR "growth falter*" OR "slow growth" OR "slowed growth"
OR malnutrition OR malnourish* OR "Severe Acute Malnutrition" OR sam OR wasting OR wasted
OR "growth restrict*" OR "growth retard*" OR "Failure to Thrive" OR ftt OR "growth disorder*" OR
"growth arrest*" OR "growth deficien*" OR "growth disturb*"))) OR ((ab:("growth failure*" OR
"growth falter*" OR "slow growth" OR "slowed growth" OR malnutrition OR malnourish* OR
"Severe Acute Malnutrition" OR sam OR wasting OR wasted OR "growth restrict*" OR "growth
retard*" OR "Failure to Thrive" OR ftt OR "growth disorder*" OR "growth arrest*" OR "growth
deficien*" OR "growth disturb*")))) AND ((mh:("Anti-Bacterial Agents")) OR ((ti:(antibiotic* OR
"anti bacterial agent*" OR antibacterial* OR "anti bacterial*" OR "bactericid*" OR "bacteriocid*"

OR "anti mycobacterial*" OR antimycobacterial*))) OR ((ab:(antibiotic* OR "anti bacterial agent*"
OR antibacterial* OR "anti bacterial*" OR "bactericid*" OR "bacteriocid*" OR "anti mycobacterial*"
OR antimycobacterial*)))) AND NOT ((mh:(animals)) AND NOT ((mh:(animals)) AND ((mh:(humans)))))

Global Index Medicus

(mh:(infant)) OR ((mh:(child))) OR ((mh:("Premature Birth"))) OR ((ti:((premature* AND (birth* OR born OR deliver*))))) OR ((ab:((premature* AND (birth* OR born OR deliver*))))) OR ((ti:((low AND (birthweight* OR "birth weight*"))))) OR ((ab:((low AND (birthweight* OR "birth weight*"))))) OR
((ti:(infant* OR infancy OR baby OR babies OR neonat* OR "neo nat*" OR newborn* OR "new born*" OR "newly born*" OR preterm OR preterms OR "pre term" OR "pre terms" OR lbw OR vlbw
OR elbw OR child* OR youth OR juvenile*))) OR ((ab:(infant* OR infancy OR baby OR babies OR
neonat* OR "neo nat*" OR newborn* OR "new born*" OR "newly born*" OR preterm OR preterms
OR "pre term" OR "pre terms" OR lbw OR vlbw OR elbw OR child* OR youth OR juvenile*)))
AND
((mh:(malnutrition)) OR ((mh:("Failure to Thrive"))) OR ((mh:("Growth Disorders"))) OR

((mh:("Severe Acute Malnutrition"))) OR ((mh:("Wasting Syndrome"))) OR
((mh:("Infant Nutrition
Disorders"))) OR ((ti:("growth failure*" OR "growth falter*" OR "slow
growth" OR "slowed growth"
OR malnutrition OR malnourish* OR "Severe Acute Malnutrition" OR sam OR wasting OR wasted
OR "growth restrict*" OR "growth retard*" OR "Failure to Thrive" OR ftt OR
"growth disorder*" OR
"growth arrest*" OR "growth deficien*" OR "growth disturb*"))) OR
((ab:("growth failure*" OR
"growth falter*" OR "slow growth" OR "slowed growth" OR malnutrition OR
malnourish* OR
"Severe Acute Malnutrition" OR sam OR wasting OR wasted OR "growth restrict*" OR
"growth
retard*" OR "Failure to Thrive" OR ftt OR "growth disorder*" OR "growth
arrest*" OR "growth
deficien*" OR "growth disturb*")))) AND ((mh:("Anti-Bacterial Agents")) OR
((ti:(antibiotic* OR
"anti bacterial agent*" OR antibacterial* OR "anti bacterial*" OR "bactericid*"
OR "bacteriocid*"
OR "anti mycobacterial*" OR antimycobacterial*))) OR ((ab:(antibiotic* OR "anti bacterial
agent*"
OR antibacterial* OR "anti bacterial*" OR "bactericid*" OR "bacteriocid*" OR
"anti mycobacterial*"
OR antimycobacterial*)))) AND NOT ((mh:(animals)) AND NOT ((mh:(animals)) AND
((mh:(humans))))) AND (collection_gim:("IMSEAR" OR "WPRIM" OR "IMEMR"
OR "AIM"))
