# Supplementary material for: Protocol for a systematic review on routine use of antibiotics for infants less than 6 months of age with growth failure/faltering
Source: BMJ Open. 2022 Jun 30;12(6):e057241. doi: 10.1136/bmjopen-2021-057241 (PMC9247651; doi:10.1136/bmjopen-2021-057241)
Supplement: Author's manuscript [file bmjopen-2021-057241.draft_revisions.pdf]

BMJ Open is committed to open peer review. As part of this commitment we make the peer review history of every article we publish publicly available.

When an article is published we post the peer reviewers' comments and the authors' responses online. We also post the versions of the paper that were used during peer review. These are the versions that the peer review comments apply to.

The versions of the paper that follow are the versions that were submitted during the peer review process. They are not the versions of record or the final published versions. They should not be cited or distributed as the published version of this manuscript.

BMJ Open is an open access journal and the full, final, typeset and author-corrected version of record of the manuscript is available on our site with no access controls, subscription charges or pay-per-view fees (http://bmjopen.bmj.com).

If you have any questions on BMJ Open's open peer review process please email info.bmjopen@bmj.com

BMJ Open

Routine antibiotics for infants less than six months of age with growth failure/faltering. A protocol for the systematic review

Journal:	*BMJ Open*
Manuscript ID	bmjopen-2021-057241
Article Type:	Protocol
Date Submitted by the Author:	09-Sep-2021
Complete List of Authors:	Imdad, Aamer; SUNY Upstate Medical University, Pediatrics<br>Chen, Fanny F; SUNY Upstate Medical University<br>François, Melissa; SUNY Upstate Medical University<br>Tanner-Smith, Emily; University of Oregon,<br>Smith, Abigail; SUNY Upstate Medical University<br>Tsistinas, Olivia; SUNY Upstate Medical University<br>Das, Jai K.; Aga Khan University, Division of Women and Child Health<br>Bhutta, Zulfiqar; The Aga Khan University, Division of Women and Child Health; SickKids
Keywords:	Nutritional support < GASTROENTEROLOGY, Paediatric gastroenterology < GASTROENTEROLOGY, NUTRITION & DIETETICS, PUBLIC HEALTH

SCHOLARONE™
Manuscripts

Routine antibiotics for infants less than six months of age with growth failure/faltering. A protocol for the systematic review

Aamer Imdad, Fanny F Chen, Melissa Francois, Emily Tanner-Smith, Abigail Smith, Olivia J Tsistinas, Jai Das, Zulfiqar Ahmed Bhutta

Authors' information

Contact Author
Aamer Imdad, MBBS, MPH
Assistant Professor of Pediatrics
Division of Pediatric Gastroenterology, Hepatology and Nutrition
SUNY Upstate Medical University, Syracuse, NY
725 Irving Street, Syracuse, NY 13210
Email: imdada@upstate.edu
Phone: 315-464-8444
Fax: 315-464-8443

Fanny Chen
SUNY Upstate Medical University
ChenF@upstate.edu

Melissa Francois
SUNY Upstate Medical University
FrancoMe@upstate.edu

Emily Tanner-Smith
University of Oregon
etanners@uoregon.edu

For peer review only

Abigail Smith
SUNY Upstate Medical University
SmithAb@upstate.edu

Olivia Tsistinas
SUNY Upstate Medical University
TsistiJO@upstate.edu

Jai Das
Aga Khan University
Jai.das@aku.edu

Zulfiqar Ahmed Bhutta
Institute for Global Health & Development, The Aga Khan University
zulfiqar.bhutta@aku.edu

Key Words: Antibiotics, wasting, malnutrition, growth failure

Word Count: 2989

For peer review only

Abstract

Introduction: Antibiotics have been used as an adjunct in treating children with severe acute malnutrition 6 to 59 months of age; however, the data for infants less than six months is scarce. The World Health Organization (WHO) recently started guideline development for preventing and treating wasting, including growth failure/faltering in infants less than six months. This systematic review commissioned by WHO aims to synthesize evidence from current literature on the effectiveness of antibiotics for infants less than six months of age with growth failure/faltering.

Methods and Analysis

We will conduct a systematic review and meta-analysis for studies that assessed the effect of antibiotics in the treatment of infants with growth faltering. We will search multiple electronic databases. We will include randomized controls trials and non-randomized studies with a control arm. The study population is infants less than six months of age with growth failure. The intervention group will be infants who received no antibiotics or antibiotics other than recommended in 2013 guidelines by WHO to treat severe acute malnutrition in children. The comparison group will be infants who received antibiotics according to the 2013 guideline by WHO. We will consider the following outcomes: Mortality, Clinical deterioration, Antimicrobial resistance, Recovery from co-morbidity, Adverse events, markers of intestinal inflammation, markers of systemic inflammation, hospital-acquired infections, Non-response. We will use the meta-analysis to pool the studies where applicable. We will use the Grading of Recommendations Assessment, Development, and Evaluation (GRADE) approach to reporting the overall evidence quality for an outcome.

Ethics and Dissemination: This is a systematic review and will not involve contact with a human subject. The findings of this review will be published in a peer-review journal and will guide the WHO's recommendation for the use of antibiotics in infants less than six months of age with growth failure.

Main Manuscript

For peer review only

Introduction

The World Health Organization (WHO)and the United Nations Children's Fund (UNICEF) estimate that nearly 14 million children suffer from severe acute malnutrition (SAM) worldwide[1]. Infants less than six months of age are particularly vulnerable to the effects of inadequate nutrition. Higher mortality rates secondary to growth failure are seen in this age group compared to older infants and children[2,3]. Despite excess mortality risk and increasing prevalence of wasting in this population, limited studies exist to guide the management of young infants with growth failure and faltering[2-4]. Malnutrition increases the risk of severe infections in children and triples the risk of mortality from pneumonia, measles, or diarrhea[5]. Therefore, the current practice for children six months to 5 years of age with SAM is to prescribe routine antibiotics, such as 5 to 7 days of amoxicillin, when they are inpatients[5]. However, current recommendations state that the same general medical care should be used for infants with SAM who are less than six months of age as infants above six months of age, even though there is limited evidence to support this recommendation for infants less than six months of age[2].

Furthermore, even though antibiotics have been shown to be effective in children 6-59 months of age with SAM, this practice in infants has the potential to harm due to recently identified risks of antibiotic use in infancy, including the diminishment of infant gut microbiome[6], future development of obesity, allergic disorders[7], and autoimmune disorders[8]. The urgency in more targeted management guidelines is further underscored by the physiological differences in renal and gastrointestinal function in infants compared to older children[8]. The WHO recently started the process of guideline development for the prevention and treatment of wasting in children, including growth failure/faltering in infants less than six months. We aim to systematically review and synthesize evidence from current literature on the effectiveness of antibiotics for infants less than six months of age with growth failure/faltering.

Review question

In infants <6 months with growth failure/faltering, what are the effects of no routine antibiotics or different* approaches (e.g., types of antibiotics, doses, etc.) compared to routine antibiotics following treatment protocols in the 2013 WHO guidelines on the outcomes of interest?

Methods

This systematic review will be conducted according to methods described in Cochrane Handbook[9] and reported using PRISMA guidelines 2020[10]. We will register the protocol on the PROSPERO website.

Types of studies to be included

We will include randomized and non-randomized controlled trials. We will consider both individual and cluster randomized trials. We will also consider non-randomised trials and cohort studies with a controlled arm. Table 1 gives the definitions of the eligible study designs. We will exclude case-control studies, case reports, case series, and commentaries.

Population

For peer review only

The population of interest is infants under six months of age with growth failure/faltering. As there is no standard definition of growth failure/faltering, we will consider author definitions. We will include studies irrespective if they are done in the community setting or the hospital setting. We will consider studies that included infants infected with HIV. We will include studies with infants born low birth weight or preterm; however, we will exclude studies on infants admitted to the neonatal intensive care units. We will exclude studies that included infants with congenital anomalies only. If a study includes infants less than six months and greater than six months of age, we will consider this study if the disaggregated data are available for infants less than six months. If the disaggregated data are not available from such a study, we will consider the study if the average age of infants is at or below 6 months of age.

Intervention

We will include all antibiotic treatments given systemically, such as amoxicillin, Augmentin, cephalosporins, and macrolides. We will include studies irrespective of dosage, frequency, duration, or route of administration; however, topical application of antibiotics will not be considered. We will consider studies if the antibiotics were given empirically at the diagnosis of growth failure or faltering. We will also consider studies if the antibiotics were given in response to a suspected infection in infants with wasting. We will exclude studies where antibiotics were given for the prevention of wasting in otherwise healthy infants. We will also exclude studies where antibiotics were given for other reasons, such as suspected serious bacterial infections in otherwise healthy infants. We will allow co-intervention such as nutritional supplementation for nutritional rehabilitation only if similar in both groups.

Comparison

The comparison groups are routine antibiotics following treatment protocols detailed in the 2013 WHO guideline[2]. We will include studies that compared no antibiotics and/or dosage and frequencies of the same drug.

Outcomes

-Mortality (Dichotomous outcome)
-Clinical deterioration [Dichotomous outcome, defined by the development of any danger signs (obstructed breathing, respiratory distress, cyanosis, shock, severe anemia, convulsion, severe dehydration, profuse watery diarrhea, vomiting, and/or impaired consciousness)]
-Antimicrobial resistance (Dichotomous outcome, as defined by authors)
-Recovery from wasting- (Dichotomous outcome, as defined by authors)
-Recovery from co-morbidity, Pneumonia (Dichotomous outcome)
-Recovery from co-morbidity, diarrhea (Dichotomous outcome)
-Recovery from any co-morbidity (Dichotomous outcome)
-Adverse events-incidence of Diarrhea (Dichotomous outcome, > 3 loose stools/24 hours)
-Markers of intestinal inflammation-fecal calprotectin (continuous outcome)
-Markers of systemic inflammation-serum C reactive protein (continuous outcome)
-Hospital-acquired infections (Dichotomous outcome)

For peer review only

-Non-response (e.g., not achieving recovery within four months of initiating treatment) (dichotomous outcome)

We will consider all the outcomes at 1, 3, and 6 months and the longest follow-up. All the primary analyses will be done at the longest follow-up.

Balance of benefits and harms
We will stratify the outcomes by benefit and harm and provide a narrative summary of the balance of benefits and harms.

Literature search
We will conduct systematic electronic queries using key terms in multiple databases, including MEDLINE via PubMed, EMBASE, Web of Science, CINAHL, Scopus, LILACS, WHO Global Index Medicus, and BIOSIS Previews. There will be no search restrictions on outcomes, publication year, publication status, or publication language. The search strategies for different databases are available in Appendix 1. The references of formerly published reviews and recently published studies will be examined for potential inclusion. We will also use The Cochrane Central Register for Controlled Trials and ISRCTN registry to identify studies currently underway. We will also search the websites of pertinent international agencies such as the WHO (including WHO's Reproductive Health Library, electronic Library of Evidence of Nutrition Actions and Global database on the Implementation of Nutrition Action), UNICEF, Global Alliance for Improved Nutrition, International Food Policy Research Institute, International Initiative for Impact Evaluation, Nutrition International, World Bank, USAID and affiliates (e.g., FANTA, SPRING), and the World Food Programme. We will search the abstracts of the major conference, such as annual pediatric academic society meetings. Finally, we will use the citation tracking function of the included studies in PubMed to look for any eligible studies.

Data extraction and synthesis
Selection of studies and data extraction
We will use a three-phased approach to screen studies for eligibility. In the first phase, two authors will independently screen study titles and abstracts yielded from search results to identify potentially eligible ones. Studies selected during this initial phase will then go through a full-text review, which constitutes the second phase. In the final phase, studies that are determined to be eligible through the full-text review will undergo data extraction. We will use the software Covidence in the screening process and create a form to store the data extracted in duplicate by two authors[11]. Two authors will screen the studies independently and compare their findings. The authors will resolve potential disagreements through discussions, and the senior author on the team may assist as needed.
We will extract characteristic data for each eligible study, including study region/country, study year, study type, intervention exposure (dose, duration, frequency), comparison, outcomes, population characteristics detailed in subgroup analysis, and risk of bias. We will also extract the information on feasibility, acceptability, equity, and resource use of the intervention and report this data in separate tables (Tables 2 and 3). We will extract the raw values for the number of events in the intervention and control group in case of dichotomous outcomes. To

For peer review only

avoid reviewer bias, we are deciding a priori the order of preference for extracting outcomes when data might be available in several formats. For randomized studies, we will prefer data that require the least manipulation by authors or inference by reviewers. We will give preference denominators in the following order for mortality data: number with the definite outcome known, number randomized, and child-years. For morbidity outcomes to which both survivors and non-survivors may have contributed data (for example, the incidence of diarrhea), we will give preference to child years/months/days, number with the definite outcome known, and number randomized.

For continuous outcomes from randomized trials, we will extract the raw values (for example, means and standard deviations) rather than calculated effect sizes (e.g., Cohen's d) and calculate the summary estimate ourselves. For denominators, we will preference the numbers with the definite outcome known and then number randomized. For non-randomized studies, we will prefer to extract the summary estimates (Mean difference or relative risk) adjusted for confounding variables.

If a study is only available in an abstract, we will contact the authors for full text. If the full text could not be obtained from any sources, we will include the abstract if sufficient details of study design and outcomes are available. We will try to find the protocol of each included study to assess the details of the methods. If the study protocol is not publicly available, we will contact the authors for the same. Suppose the results of the randomized trial are published in more than one report. In that case, we will consider all the publications related to that study as one study but extract the data from all relevant publications.

Assessment of risk of bias in included studies

We will evaluate the study risk of bias with the Cochrane risk of bias (ROB 2.0) and Cochrane ROBINS-I for randomized controlled trials and non-randomized studies of interventions, respectively[12,13]. Two authors will assess the risk of bias separately and compare their findings and reach a consensus. In the case of conflicts, a discussion will be held to resolve the conflict. A senior author will be consulted if a consensus could be reached between the two primary authors.

The risk of bias assessment according to ROB-2 and ROBINS-I is done for each outcome and not for a particular study[12,13]. This risk of bias assessment is different from earlier practice where a study was assessed for risk of bias, and the same assessment was applied to all the outcomes extracted from that study[9]. We will summarize the risk of bias for each domain (such as sequence generation, allocation concealment, etc.) and the overall risk of bias from the study for a particular outcome. The risk of bias will be summarized as high, low, or some concerns. We will present the risk of bias assessment in the form of graphs.

Data Synthesis

We will report review findings both qualitatively and quantitatively. A narrative synthesis will be used to report the characteristics of all included studies and their results. A random-effects meta-analysis will be employed when at least two studies possess sufficient clinical and methodological uniformity for synthesis. If quantitative synthesis is not appropriate, results will be described according to the standard guidelines of narrative synthesis[14]. The software

For peer review only

RevMan will be utilized for statistical analysis[15]. Dichotomous outcomes will be assessed using relative risk (RR) effect sizes and presented with 95% confidence intervals (CI). The continuous outcomes will be combined to obtain a standardized mean difference and reported with a 95 % CI. We will present the meta-analysis results in the form of forest plots and examine their statistical heterogeneity and inconsistency.

We will include the data from randomized controlled trials (RCT) on an intention to treat analysis basis. If an RCT does not report an intention to treat analysis, we will generate our own intention to treat analysis by using the raw values extracted from the trial. Suppose an RCT does not report the raw values but reports the summary estimate, such as relative risk. In that case, we will include the summary estimate using the generic inverse variance weighting method of meta-analysis. For observational studies, we will prefer to use a summary estimate that was adjusted for confounding factors. If a non-randomized study reports an odd ratio as the summary estimate, we will calculate the relative risk if the raw data are available. If the raw data are not available to calculate a relative risk, we will pool the odd ratio if the large study sample size. We will do the sensitivity analysis by excluding such studies to see if it has any detrimental effect on the final summary estimate of the pooled analysis.

We plan to pool the data separately from observational studies and randomized studies. Suppose the number of studies is small (less than 10). In that case, we may pool both the study designs in the same analysis but do a sensitivity analysis by excluding the observational studies.

We will consider the following two comparisons.

1) No routine antibiotics compared to routine antibiotics following treatment protocols in the 2013 WHO guidelines

2) Different approaches to the use of antibiotics (e.g., types of antibiotics, doses, etc.) compared to the routine antibiotics following treatment protocols in the 2013 WHO guidelines

Studies with missing data

We will record missing data during the data extraction process. We will use imputations of missing data over recording the data as missing. If data are missing for some cases or dropouts are not reported, we will contact the study authors requesting the complete data. If a study does imputations for the missing data, we will use the imputed data. If a study does not report the raw values, we will extract the summary estimates such as relative risk or risk ratio. If a study does not report mean but median, we will convert the median to mean based on the Cochrane Handbook of Systematic Reviews methods[9]. Suppose the SD is not available for a mean, and the value cannot be calculated from available data such as standard error, confidence interval, or the p-value. In that case, we will contact the authors for additional information. If the SD is not available from any source for a mean, we will use the SD from a similar study. If the data are presented only in graphs, we will extract the information to the best possible approximation.

For peer review only - http://bmjopen.bmj.com/site/about/guidelines.xhtml

Assessment of heterogeneity

We will analyze statistical heterogeneity in the pooled data using Tau^2, χ^2, and I^2 statistics. We will also assess statistical heterogeneity through visual inspection of forest plots, utilizing the χ^2 test (assessing the p-value) and calculating the Tau^2 and I^2 statistics. We will consider it significant statistical heterogeneity when the p-value is less than 0.1, the I2 value exceeds 50%, and the inspection of forest plots shows substantial variability in the effect of the intervention. Finally, we will perform subgroup analysis to identify reasons for eligible statistical heterogeneity.

Assessment of reporting bias

We will assess publication bias of small studies using funnel plots and regression tests for funnel plot asymmetry when the meta-analysis includes at least ten studies.

Subgroup analysis and investigation of heterogeneity

- By different types/definitions of growth failure/faltering
- Age at presentation [newborn (0-28 days), 1-3 months, 4-6 months]
- Gestational age: Preterm birth (< 37 weeks) vs. full-term birth (> 37 weeks)
- Birth weight: Low birth weight (<2500 g) vs. Normal birth weight (> 2500 g)
- HIV exposure: Studies with Participants exposed to HIV VS. Studies with no HIV exposure
- Presentation: Participants with Oedema vs. Patricians with no oedema
- Co-morbidities: With or without co-morbidities
- Nutrition: Babies Breastfeeding or non-breastfed babies
- Location of the treatment: inpatient or outpatient/community
- Dose of antibiotics
- Duration of antibiotics: 7 days vs. > 7 days
- Type of antibiotics

The subgroup analyses above will be based on individual-level data. We will try to extract the information from individual studies as available. If the information is not available from the published studies, we will write to the authors to get the required data.

Sensitivity analysis

We will complete sensitivity analysis by removing studies with a high risk of bias. We will compare results from random versus fixed-effect meta-analysis models.

Rating of overall quality of evidence

The Grading of Recommendations Assessment, Development, and Evaluation (GRADE) approach will be used to evaluate the overall certainty of evidence using the software GRADEpro[16]. The GRADE approach is a comprehensive framework used to assess the overall certainty of the evidence for an outcome utilizing study characteristics such as study design, inconsistency, indirectness of evidence, risk of bias, publication bias, and imprecision estimates. We will include the GRADE assessment results in a GRADE evidence profile that contains

For peer review only

certainty ratings, including very low, low, moderate, or high based on the evidence across studies for primary outcomes (table 4).

Amendments
We will conduct literature searches, screening of titles, selection of studies, data extraction, and analysis according to the plan aforementioned in the protocol. We will report any additional analysis performed in the methods section if such occurs.

Patient and public involvement
No patient or public involvement was considered in the preparation of this protocol.

Consent for publication
The authors give consent for the publication of the review.

Availability of data and materials
We will provide data available for review by the editors and peer reviewers and provide raw data for the general public on request.

Ethics and dissemination
This review is intended as background work for WHO's guide to the therapeutic use of antibiotics in malnourished infants under six months. Considering this is a systematic review, there will be no direct contact with the patients included in this study. The findings stemming from this systematic review will be distributed to relevant populations, including patients, caregivers, and healthcare workers.

Funding
This work is funded by World Health Organization grant no 202725572. WHO also provided technical support for this work.

Acknowledgements
We are very thankful to Allison Daniel, Jaden BENDABENDA and Zita WEISE PRINZO for their input to improve this protocol.

References
1. United Nations Children’s Fund (UNICEF), World Health Organization, International Bank for Reconstruction and Development/The World Bank. Levels and trends in child malnutrition: Key Findings of the 2020 Edition of the Joint Child Malnutrition Estimates. Geneva: World Health Organization. Licence: CC BY-NC-SA 3.0 IGO. 2020.
2. WHO. Guideline: Updates on the management of severe acute malnutrition in infants and children. Geneva: World Health Organization. 2013.
3. Kerac M, Blencowe H, Grijalva-Eternod C, et al. prevalence of wasting among under 6-month-old infants in developing countries and implications of new case definitions using WHO growth standards: a secondary data analysis. Arch Dis Child 2011;96:1008-13.

For peer review only

4. Patwari AK, Kumar S, Beard J. Undernutrition among infants less than 6 months of age: an underestimated public health problem in India. Matern Child Nutr 2015;11:119-26.
5. Alcoba G, Kerac M, Breysse S, et al. Do children with uncomplicated severe acute malnutrition need antibiotics? A systematic review and meta-analysis. PLoS One 2013;8:e53184.
6. Stam J, van Stuijvenberg M, Gruber C, et al. Antibiotic use in infants in the first year of life in five European countries. Acta Paediatr 2012;101:929-34.
7. Droste JH, Wieringa MH, Weyler JJ, Nelen VJ, Vermeire PA, Van Bever HP. Does the use of antibiotics in early childhood increase the risk of asthma and allergic disease? Clin Exp Allergy 2000;30:1547-53.
8. Trasande L, Blustein J, Liu M, Corwin E, Cox LM, Blaser MJ. Infant antibiotic exposures and early-life body mass. Int J Obes (Lond) 2013;37:16-23.
9. Higgins JPT TJ, Chandler J, Cumpston M, Li T, Page MJ, Welch VA (editors). Cochrane Handbook for Systematic Reviews of Interventions Version 6.2 Available from www.training.cochrane.org/handbook. 2021.
10. Page MJ, McKenzie JE, Bossuyt PM, et al. The PRISMA 2020 statement: an updated guideline for reporting systematic reviews. BMJ 2021;372:n71.
11. Covidence systematic review software. veritas health innovation. Melbourne, Australia: www.covidence.org; 2021.
12. Sterne JAC, Savovic J, Page MJ, et al. RoB 2: a revised tool for assessing risk of bias in randomised trials. BMJ 2019;366:l4898.
13. Sterne JA, Hernan MA, Reeves BC, et al. ROBINS-I: a tool for assessing risk of bias in non-randomised studies of interventions. BMJ 2016;355:i4919.
14. Campbell M, McKenzie JE, Sowden A, et al. synthesis without meta-analysis (SWiM) in systematic reviews: reporting guideline. BMJ 2020;368:l6890.
15. Review Manager (RevMan). 5.3 ed. Copenhagen: The Nordic Cochrane Center TCC; 2014.
16. GRADEpro GDT: GRADEpro guideline development tool. McMaster University: Evidence Prime, Inc; 2020.
17. Guyatt G, Oxman AD, Akl EA, et al. GRADE guidelines: 1. Introduction-GRADE evidence profiles and summary of findings tables. J Clin Epidemiol 2011;64:383-94.

For peer review only

Table 1: Suggested study designs for inclusion in the review

Suggested terms	Definition	Notes
Randomised controlled trial (RCT) OR randomised trial	An experimental study in which people are allocated to different interventions using methods that are random.	We will consider both individual and cluster randomized trials. We also consider the factorial design trials
Non-randomised controlled trial (NRCT) OR non-randomised trial	An experimental study in which people are allocated to different interventions using methods that are not random.	We will use consistent terminology and avoid using the term quasi-experimental as it might have different meanings in different settings. We will use the ROBINS-1 for risk of bias assessment for this study design
Controlled before-after study (CBA)	A study in which observations are made before and after the implementation of an intervention, both in a group that receives the intervention and in a control group that does not.	We will require two minimum criteria for the inclusion of CBAs.<br>-Data Collection: We will include CBAs if the data for the intervention and control groups were collected prospectively in the same time frame<br>-Choice of Control: We will include CBAs that include a control at a second site to avoid contamination of the intervention to the control group if the settings and populations are the same for the intervention and control groups.

This table is modified from:
https://epoc.cochrane.org/sites/epoc.cochrane.org/files/public/uploads/EPOC%20Study%20Designs%20About.pdf

Table 2: Sample table to report the qualitative data from the included studies

Study	Study population	Intervention studied	Feasibility of intervention	Acceptability of intervention	Considerations for Equity

For peer review only

Table 3: Sample table for a description of qualitative data on resource use

**Study**	**Intervention studies**	**Resources used for production**	**Resources used for administration of the intervention**

Table 4: Description of methods for grading of overall evidence

Study Design	Quality of Evidence	Lower Certainty Score if	Higher certainty score if
Randomized trial	High	Risk of Bias<br>-1 Serious<br>-2 Very serious<br><br>Inconsistency<br>-1 Serious<br>-2 Very serious<br><br>Indirectness<br>-1 Serious<br>-2 Very serious<br><br>Imprecision<br>-1 Serious<br>-2 Very serious<br><br>Publication Bias<br>-1 Likely<br>-2 Very likely	Large effect<br>+1 Large<br>+2 Very large<br><br>Dose-response<br>+1 evidence of a gradient<br><br>All plausible confounding would:<br>+1 Reduce a demonstrated effect<br><br>+1 Suggest a spurious effect when results show no effect
	Moderate		
Observational study	Low		
	Very Low		

* In the GRADE approach, randomized controlled trials (RCTs) start as high-quality evidence and observational studies as low-quality evidence supporting estimates of intervention effects. Five factors may lead to rating down the quality of evidence, and three factors may lead to rating up. Ultimately, the quality of evidence for each outcome falls into one of four categories, from high to very low. GRADE is "outcome centric": rating is made for each outcome, and quality may differ indeed, is likely to differ from one outcome to another within a single study and across a body of evidence. [17]

For peer review only - http://bmjopen.bmj.com/site/about/guidelines.xhtml

Article Summary: Strengths and Limitations

- We will search several electronic databases and include randomized and non-randomized studies.

-To authors will screen the titles and abstracts extract the data from included studies duplication. We will assess the risk of bias for each outcome and use the grading of recommendation, assessment, development and evaluation (GRADE) criteria to assess the overall quality of evidence

-We will contact the meta-analysis if the data is available for more than one study for an outcome and there is clinical and methodological homogeneity in the included studies

-All the included studies may not report the data for all the outcomes mentioned in this review which will be a limitation of the study.

For peer review only

Appendix 1

Search Strategies for Growth Failure AND Antibiotics

PubMed

(("Infant"[MeSH Terms] OR "Child"[MeSH Terms:noexp] OR "Premature Birth"[MeSH Terms] OR ("infant*"[Title/Abstract] OR "infancy"[Title/Abstract] OR "baby"[Title/Abstract] OR "babies"[Title/Abstract] OR "neonat*"[Title/Abstract] OR "neo nat*"[Title/Abstract] OR "newborn*"[Title/Abstract] OR "new born*"[Title/Abstract] OR "newly born*"[Title/Abstract] OR "preterm"[Title/Abstract] OR "preterms"[Title/Abstract] OR "pre term"[Title/Abstract] OR "pre terms"[Title/Abstract] OR "lbw"[Title/Abstract] OR "vlbw"[Title/Abstract] OR "elbw"[Title/Abstract] OR "child*"[Title/Abstract] OR "youth"[Title/Abstract] OR "juvenile*"[Title/Abstract]) OR ("premature*"[Title/Abstract] AND ("birth*"[Title/Abstract] OR "born"[Title/Abstract] OR "deliver*"[Title/Abstract])) OR ("low"[Title/Abstract] AND ("birthweight*"[Title/Abstract] OR "birth weight*"[Title/Abstract]))) AND ("Malnutrition"[MeSH Terms] OR "Failure to Thrive"[MeSH Terms] OR "Growth Disorders"[MeSH Terms:noexp] OR "Severe Acute Malnutrition"[MeSH Terms] OR "Wasting Syndrome"[MeSH Terms:noexp] OR "Infant Nutrition Disorders"[MeSH Terms:noexp] OR ("growth failure*"[Title/Abstract] OR "growth falter*"[Title/Abstract] OR "slow growth"[Title/Abstract] OR "slowed growth"[Title/Abstract] OR "Malnutrition"[Title/Abstract] OR "malnourish*"[Title/Abstract] OR "Severe Acute Malnutrition"[Title/Abstract] OR "SAM"[Title/Abstract] OR "wasting"[Title/Abstract] OR "wasted"[Title/Abstract] OR "growth restrict*"[Title/Abstract] OR "growth retard*"[Title/Abstract] OR "Failure to Thrive"[Title/Abstract] OR "FTT"[Title/Abstract] OR "growth disorder*"[Title/Abstract] OR "growth arrest*"[Title/Abstract] OR "growth deficien*"[Title/Abstract] OR "growth disturb*"[Title/Abstract])) AND ("Anti-Bacterial Agents"[Pharmacological Action] OR "Anti-Bacterial Agents"[MeSH Terms] OR ("antibiotic*"[Title/Abstract] OR "anti bacterial agent*"[Title/Abstract] OR "antibacterial*"[Title/Abstract] OR "anti bacterial*"[Title/Abstract] OR "bactericid*"[Title/Abstract] OR "bacteriocid*"[Title/Abstract] OR "anti mycobacterial*"[Title/Abstract] OR "antimycobacterial*"[Title/Abstract]))) NOT ("Animals"[MeSH Terms] NOT ("Animals"[MeSH Terms] AND "Humans"[MeSH Terms]))

CINAHL

S1 (MH "Infant+")
S2 (MH "Child")
S3 (MH "Childbirth, Premature")
S4 TI (infant* OR infancy OR baby OR babies OR neonat* OR "neo nat*" OR newborn* OR "new

For peer review only

born*" OR "newly born*" OR preterm OR preterms OR "pre term" OR "pre terms" OR lbw OR vlbw
OR elbw OR child* OR youth OR juvenile*)
S5 AB (infant* OR infancy OR baby OR babies OR neonat* OR "neo nat*" OR newborn* OR "new born*" OR "newly born*" OR preterm OR preterms OR "pre term" OR "pre terms" OR lbw OR vlbw
OR elbw OR child* OR youth OR juvenile*)
S6 TI (premature* N2 (birth* OR born OR deliver*))
S7 AB (premature* N2 (birth* OR born OR deliver*))
S8 TI (low N2 (birthweight* OR "birth weight*"))
S9 AB (low N2 (birthweight* OR "birth weight*"))
S10 S1 OR S2 OR S3 OR S4 OR S5 OR S6 OR S7 OR S8 OR S9

S11 (MH "Malnutrition")
S12 (MH "Failure to Thrive")
S13 (MH "Growth Disorders")
S14 (MH "Wasting Syndrome")
S15 (MH “Infant Nutrition Disorders”)
S16 TI ((grow*) N2 (fail* OR falter* OR slow* OR restrict* OR retard* OR deficien* OR arrest* OR disturb* OR disorder*))
S17 AB ((grow*) N2 (fail* OR falter* OR slow* OR restrict* OR retard* OR deficien* OR arrest* OR disturb* OR disorder*))
S18 TI ((fail*) W2 (thrive))
S19 AB ((fail*) W2 (thrive))
S20 TI (Malnutrition OR malnourish* OR "Severe Acute Malnutrition" OR SAM OR wasting OR wasted OR FTT)
S21 AB (Malnutrition OR malnourish* OR "Severe Acute Malnutrition" OR SAM OR wasting OR wasted OR FTT)
S22 S11 OR S12 OR S13 OR S14 OR S15 OR S16 OR S17 OR S18 OR S19 OR S20 OR S21
S23 (MH "Antibiotics+")
S24 TI (antibiotic* OR "anti bacterial*" OR "antibacterial*" OR bactericid*OR bacteriocid* OR "anti
mycobacterial*" OR "antimycobacterial*")
S25 AB (antibiotic* OR "anti bacterial*" OR "antibacterial*" OR bactericid* OR bacteriocid* OR
"anti mycobacterial*" OR "antimycobacterial*")
S26 S23 OR S24 OR S25
S27 (MH "Animals") NOT ((MH "Animals") AND (MH "Human"))
S28 S10 AND S22 AND S26
S29 S28 NOT S27
S30 S28 NOT S27 Exclude Medline Records

Embase

#1'infant'/exp OR 'newborn'/exp OR 'prematurity'/exp OR 'low birth weight'/exp OR 'very low birth
weight'/exp OR 'extremely low birth weight'/exp OR 'juvenile'/de OR 'child'/de
#2 infant*:ti,ab OR infancy:ti,ab OR baby:ti,ab OR babies:ti,ab OR neonat*:ti,ab OR 'neo nat*':ti,ab OR newborn*:ti,ab OR 'new born*':ti,ab OR 'newly born*':ti,ab OR preterm:ti,ab OR preterms:ti,ab
OR 'pre term':ti,ab OR 'pre terms':ti,ab OR lbw:ti,ab OR vlbw:ti,ab OR elbw:ti,ab OR child*:ti,ab OR
youth:ti,ab OR juvenile*:ti,ab
#3 (premature* NEAR/2 (birth* OR born OR deliver*)):ti,ab

For peer review only

#4 (low NEAR/2 (birthweight* OR 'birth weight*')):ti,ab
#5 #1 OR #2 OR #3 OR #4

#6 'malnutrition'/exp OR 'failure to thrive'/exp OR 'growth disorder'/de OR 'wasting syndrome'/de
OR 'growth retardation'/de
#7(grow* NEAR/2 (fail* OR falter* OR slow* OR restrict* OR retard* OR deficien* OR arrest* OR disturb* OR disorder*)):ti,ab
#8 (fail* NEXT/2 thrive):ti,ab
#9 malnutrition:ti,ab OR malnourish*:ti,ab OR 'severe acute malnutrition':ti,ab OR sam:ti,ab OR wasting:ti,ab OR wasted:ti,ab OR ftt:ti,ab
#10 #6 OR #7 OR #8 OR #9
#11'antibiotic agent'/exp OR 'bactericide'/de OR 'antimycobacterial agent'/de
#12 antibiotic*:ti,ab OR 'anti bacterial*':ti,ab OR 'antibacterial*':ti,ab OR bactericid*:ti,ab OR bacteriocid*:ti,ab OR antimycobacterial*:ti,ab OR 'anti mycobacterial*':ti,ab
#13 #11 OR #12
#14 #5 AND #10 AND #13
#15 #14 NOT ('animals'/exp NOT 'humans'/exp)
#16 #15 NOT [medline]/lim

CENTRAL

#1 MeSH descriptor: [Infant] explode all trees
#2 MeSH descriptor: [Child] this term only
#3 MeSH descriptor: [Premature Birth] explode all trees
#4 infant*:ti,ab OR infancy:ti,ab OR baby:ti,ab OR babies:ti,ab OR neonat*:ti,ab OR newborn*:ti,ab OR preterm:ti,ab OR preterms:ti,ab OR lbw:ti,ab OR vlbw:ti,ab OR elbw:ti,ab OR child*:ti,ab OR youth:ti,ab OR juvenile*:ti,ab
#5 (neo NEXT nat* OR new NEXT born* OR newly NEXT born* OR pre NEXT term OR pre NEXT terms):ti,ab
#6 (premature* NEAR/2 (birth* OR born OR deliver*)):ti,ab
#7 (low NEAR/2 (birthweight* OR birth NEXT weight*)):ti,ab
#8 #1 OR #2 OR #3 OR #4 OR #5 OR #6 OR #7
#9 MeSH descriptor: [Malnutrition] explode all trees
#10 MeSH descriptor: [Failure to Thrive] explode all trees
#11 MeSH descriptor: [Growth Disorders] this term only
#12 MeSH descriptor: [Severe Acute Malnutrition] explode all trees
#13 MeSH descriptor: [Wasting Syndrome] this term only
#14 MeSH descriptor: [Infant Nutrition Disorders] this term only
#15 ((grow*) NEAR/2 (fail* OR falter* OR slow* OR restrict* OR retard* OR deficien* OR arrest* OR disturb* OR disorder*)):ti,ab

#16 ((fail*) NEXT/2 (thrive)):ti,ab
#17 (severe NEXT acute NEXT malnutrition):ti,ab
#18 malnutrition:ti,ab OR malnourish*:ti,ab OR sam:ti,ab OR wasting:ti,ab OR wasted:ti,ab OR ftt:ti,ab
#19 #9 OR #10 OR #11 OR #12 OR #13 OR #14 OR #15 OR #16 OR #17 OR #18
#20 MeSH descriptor: [Anti-Bacterial Agents] explode all trees
#21 antibiotic*:ti,ab OR antibacterial*:ti,ab OR bactericid*:ti,ab OR bacteriocid*:ti,ab OR antimycobacterial*:ti,ab
#22 (anti NEXT bacterial*):ti,ab
#23 (anti NEXT mycobacterial*):ti,ab
#24 #20 OR #21 OR #22 OR #23
#25 #8 AND #19 AND #24
#26 MeSH descriptor: [Animals] explode all trees

For peer review only

#27 MeSH descriptor: [Humans] explode all trees
#28 (#26 NOT (#26 AND #27))
#29 #25 NOT #28
#30 "accession number" NEAR pubmed
#31 #29 NOT #30

Scopus

((TITLE-ABS (infant* OR infancy OR baby OR babies OR neonat* OR "neo nat*" OR newborn* OR "new born*" OR "newly born*" OR preterm OR preterms OR "pre term" OR
"pre terms" OR lbw OR vlbw OR elbw OR child* OR youth OR juvenile*)) OR (TITLE-ABS (low W/2 (birthweight* OR "birth weight*"))) OR (TITLE-ABS (premature* W/2 (birth* OR born OR deliver*)))) AND ((TITLE-ABS ((grow*) W/2 (fail* OR falter* OR slow* OR restrict* OR retard* OR deficien* OR arrest* OR disturb* OR disorder*))) OR (TITLE-ABS ((fail*) PRE/2 (thrive))) OR (TITLE-ABS (malnutrition OR malnourish* OR "Severe Acute Malnutrition" OR sam OR wasting OR wasted OR ftt))) AND (TITLE-ABS (antibiotic* OR "anti bacterial*" OR "antibacterial*" OR bactericid* OR bacteriocid* OR "anti mycobacterial*"
OR "antimycobacterial*")) AND NOT INDEX (medline)

Web of Science

#1 TI=(infant* OR infancy OR baby OR babies OR neonat* OR "neo nat*" OR newborn* OR "new born*" OR "newly born*" OR preterm OR preterms OR "pre term" OR "pre terms" OR
lbw OR vlbw OR elbw OR child* OR youth OR juvenile*)
#2 AB=(infant* OR infancy OR baby OR babies OR neonat* OR "neo nat*" OR newborn* OR "new born*" OR "newly born*" OR preterm OR preterms OR "pre term" OR "pre terms" OR
lbw OR vlbw OR elbw OR child* OR youth OR juvenile*)
#3 TI=(premature* NEAR/2 (birth* OR born OR deliver*))

#4 AB=(premature* NEAR/2 (birth* OR born OR deliver*))
#5 TI=(low NEAR/2 (birthweight* OR "birth weight*"))
#6 AB=(low NEAR/2 (birthweight* OR "birth weight*"))
#7 #6 OR #5 OR #4 OR #3 OR #2 OR #1
#8 TI=((grow*) NEAR/2 (fail* OR falter* OR slow* OR restrict* OR retard* OR deficien* OR arrest* OR disturb* OR disorder*))
#9 AB=((grow*) NEAR/2 (fail* OR falter* OR slow* OR restrict* OR retard* OR deficien* OR arrest* OR disturb* OR disorder*))
#10 TI=((fail*) NEAR/2 (thrive))
#11 AB=((fail*) NEAR/2 (thrive))
#12 TI =(Malnutrition OR malnourish* OR "Severe Acute Malnutrition" OR SAM OR wasting OR wasted OR FTT)
#13 AB=(Malnutrition OR malnourish* OR "Severe Acute Malnutrition" OR SAM OR wasting OR wasted OR FTT)
#14 #13 OR #12 OR #11 OR #10 OR #9 OR #8
#15 TI=(antibiotic* OR "anti bacterial*" OR "antibacterial*" OR bactericid* OR bacteriocid* OR "anti
mycobacterial*" OR "antimycobacterial*")
#16 AB=(antibiotic* OR "anti bacterial*" OR "antibacterial*" OR bactericid* OR bacteriocid* OR
"anti mycobacterial*" OR "antimycobacterial*")
#17 #16 OR #15
#18 #17 AND #14 AND #7

LILACS

For peer review only

(mh:(infant)) OR ((mh:(child))) OR ((mh:("Premature Birth"))) OR ((ti:((premature* AND (birth* OR born OR deliver*))))) OR ((ab:((premature* AND (birth* OR born OR deliver*))))) OR ((ti:((low AND (birthweight* OR "birth weight*"))))) OR ((ab:((low AND (birthweight* OR "birth weight*"))))) OR
((ti:(infant* OR infancy OR baby OR babies OR neonat* OR "neo nat*" OR newborn* OR "new born*" OR "newly born*" OR preterm OR preterms OR "pre term" OR "pre terms" OR lbw OR vlbw
OR elbw OR child* OR youth OR juvenile*))) OR ((ab:(infant* OR infancy OR baby OR babies OR neonat* OR "neo nat*" OR newborn* OR "new born*" OR "newly born*" OR preterm OR preterms
OR "pre term" OR "pre terms" OR lbw OR vlbw OR elbw OR child* OR youth OR juvenile*)))
AND
((mh:(malnutrition)) OR ((mh:("Failure to Thrive"))) OR ((mh:("Growth Disorders"))) OR ((mh:("Severe Acute Malnutrition"))) OR ((mh:("Wasting Syndrome"))) OR ((mh:("Infant Nutrition
Disorders"))) OR ((ti:("growth failure*" OR "growth falter*" OR "slow growth" OR "slowed growth"
OR malnutrition OR malnourish* OR "Severe Acute Malnutrition" OR sam OR wasting OR wasted
OR "growth restrict*" OR "growth retard*" OR "Failure to Thrive" OR ftt OR "growth disorder*" OR
"growth arrest*" OR "growth deficien*" OR "growth disturb*"))) OR ((ab:("growth failure*" OR
"growth falter*" OR "slow growth" OR "slowed growth" OR malnutrition OR malnourish* OR
"Severe Acute Malnutrition" OR sam OR wasting OR wasted OR "growth restrict*" OR "growth
retard*" OR "Failure to Thrive" OR ftt OR "growth disorder*" OR "growth arrest*" OR "growth
deficien*" OR "growth disturb*")))) AND ((mh:("Anti-Bacterial Agents")) OR ((ti:(antibiotic* OR
"anti bacterial agent*" OR antibacterial* OR "anti bacterial*" OR "bactericid*" OR "bacteriocid*"

OR "anti mycobacterial*" OR antimycobacterial*))) OR ((ab:(antibiotic* OR "anti bacterial agent*"
OR antibacterial* OR "anti bacterial*" OR "bactericid*" OR "bacteriocid*" OR "anti mycobacterial*"
OR antimycobacterial*)))) AND NOT ((mh:(animals)) AND NOT ((mh:(animals)) AND ((mh:(humans)))))

Global Index Medicus

(mh:(infant)) OR ((mh:(child))) OR ((mh:("Premature Birth"))) OR ((ti:((premature* AND (birth* OR born OR deliver*))))) OR ((ab:((premature* AND (birth* OR born OR deliver*))))) OR ((ti:((low AND (birthweight* OR "birth weight*"))))) OR ((ab:((low AND (birthweight* OR "birth weight*"))))) OR
((ti:(infant* OR infancy OR baby OR babies OR neonat* OR "neo nat*" OR newborn* OR "new born*" OR "newly born*" OR preterm OR preterms OR "pre term" OR "pre terms" OR lbw OR vlbw
OR elbw OR child* OR youth OR juvenile*))) OR ((ab:(infant* OR infancy OR baby OR babies OR neonat* OR "neo nat*" OR newborn* OR "new born*" OR "newly born*" OR preterm OR preterms
OR "pre term" OR "pre terms" OR lbw OR vlbw OR elbw OR child* OR youth OR juvenile*)))
AND
((mh:(malnutrition)) OR ((mh:("Failure to Thrive"))) OR ((mh:("Growth Disorders"))) OR

For peer review only

((mh:("Severe Acute Malnutrition"))) OR ((mh:("Wasting Syndrome"))) OR ((mh:("Infant Nutrition Disorders"))) OR ((ti:("growth failure*" OR "growth falter*" OR "slow growth" OR "slowed growth" OR malnutrition OR malnourish* OR "Severe Acute Malnutrition" OR sam OR wasting OR wasted OR "growth restrict*" OR "growth retard*" OR "Failure to Thrive" OR ftt OR "growth disorder*" OR "growth arrest*" OR "growth deficien*" OR "growth disturb*"))) OR ((ab:("growth failure*" OR "growth falter*" OR "slow growth" OR "slowed growth" OR malnutrition OR malnourish* OR "Severe Acute Malnutrition" OR sam OR wasting OR wasted OR "growth restrict*" OR "growth retard*" OR "Failure to Thrive" OR ftt OR "growth disorder*" OR "growth arrest*" OR "growth deficien*" OR "growth disturb*")))) AND ((mh:("Anti-Bacterial Agents")) OR ((ti:(antibiotic* OR "anti bacterial agent*" OR antibacterial* OR "anti bacterial*" OR "bactericid*" OR "bacteriocid*" OR "anti mycobacterial*" OR antimycobacterial*))) OR ((ab:(antibiotic* OR "anti bacterial agent*" OR antibacterial* OR "anti bacterial*" OR "bactericid*" OR "bacteriocid*" OR "anti mycobacterial*" OR antimycobacterial*)))) AND NOT ((mh:(animals)) AND NOT ((mh:(animals)) AND ((mh:(humans))))) AND (collection_gim:("IMSEAR" OR "WPRIM" OR "IMEMR" OR "AIM"))

For peer review only

Routine antibiotics for infants less than six months of age with growth failure/faltering. A protocol for the systematic review

Aamer Imdad, Fanny F Chen, Melissa Francois, Emily Tanner-Smith, Abigail Smith, Olivia J Tsistinas, Jai Das, Zulfiqar Ahmed Bhutta

PRISMA-P (Preferred Reporting Items for Systematic review and Meta-Analysis Protocols) 2015 checklist: recommended items to address in a systematic review protocol*

Section and topic	Item No	Checklist item	This manuscript
**ADMINISTRATIVE INFORMATION**			
Title:			
Identification	1a	Identify the report as a protocol of a systematic review	Done
Update	1b	If the protocol is for an update of a previous systematic review, identify as such	
Registration	2	If registered, provide the name of the registry (such as PROSPERO) and registration number	Application pending
Authors:			
Contact	3a	Provide name, institutional affiliation, e-mail address of all protocol authors; provide physical mailing address of corresponding author	Done
Contributions	3b	Describe contributions of protocol authors and identify the guarantor of the review	
Amendments	4	If the protocol represents an amendment of a previously completed or published protocol, identify as such and list changes; otherwise, state plan for documenting important protocol amendments	Done
Support:			
Sources	5a	Indicate sources of financial or other support for the review	
Sponsor	5b	Provide name for the review funder and/or sponsor	done
Role of sponsor or funder	5c	Describe roles of funder(s), sponsor(s), and/or institution(s), if any, in developing the protocol	
**INTRODUCTION**			
Rationale	6	Describe the rationale for the review in the context of what is already known	
Objectives	7	Provide an explicit statement of the question(s) the review will address with reference to participants, interventions, comparators, and outcomes (PICO)	Done
**METHODS**			

For peer review only

Eligibility criteria	8	Specify the study characteristics (such as PICO, study design, setting, time frame) and report characteristics (such as years considered, language, publication status) to be used as criteria for eligibility for the review	Done
Information sources	9	Describe all intended information sources (such as electronic databases, contact with study authors, trial registers or other grey literature sources) with planned dates of coverage	Done
Search strategy	10	Present draft of search strategy to be used for at least one electronic database, including planned limits, such that it could be repeated	Done
Study records:			
Data management	11a	Describe the mechanism(s) that will be used to manage records and data throughout the review	
Selection process	11b	State the process that will be used for selecting studies (such as two independent reviewers) through each phase of the review (that is, screening, eligibility and inclusion in meta-analysis)	
Data collection process	11c	Describe planned method of extracting data from reports (such as piloting forms, done independently, in duplicate), any processes for obtaining and confirming data from investigators	
Data items	12	List and define all variables for which data will be sought (such as PICO items, funding sources), any pre-planned data assumptions and simplifications	
Outcomes and prioritization	13	List and define all outcomes for which data will be sought, including prioritization of main and additional outcomes, with rationale	Done
Risk of bias in individual studies	14	Describe anticipated methods for assessing risk of bias of individual studies, including whether this will be done at the outcome or study level, or both; state how this information will be used in data synthesis	Done
Data synthesis	15a	Describe criteria under which study data will be quantitatively synthesised	
	15b	If data are appropriate for quantitative synthesis, describe planned summary measures, methods of handling data and methods of combining data from studies, including any planned exploration of consistency (such as I^2, Kendall's τ)	Done
	15c	Describe any proposed additional analyses (such as sensitivity or subgroup analyses, meta-regression)	
	15d	If quantitative synthesis is not appropriate, describe the type of summary planned	
Meta-bias(es)	16	Specify any planned assessment of meta-bias(es) (such as publication bias across studies, selective reporting within studies)	Done
Confidence in cumulative evidence	17	Describe how the strength of the body of evidence will be assessed (such as GRADE)	Done

For peer review only

BMJ Open

A Protocol for a Systematic Review on Routine Use of Antibiotics for Infants Less Than Six Months of Age with Growth Failure/Faltering

Journal:	*BMJ Open*
Manuscript ID	bmjopen-2021-057241.R1
Article Type:	Protocol
Date Submitted by the Author:	23-May-2022
Complete List of Authors:	Imdad, Aamer; SUNY Upstate Medical University, Pediatrics<br>Chen, Fanny F; SUNY Upstate Medical University<br>François, Melissa; SUNY Upstate Medical University<br>Tanner-Smith, Emily; University of Oregon,<br>Smith, Abigail; SUNY Upstate Medical University<br>Tsistinas, Olivia; SUNY Upstate Medical University<br>Das, Jai K.; Aga Khan University, Division of Women and Child Health<br>Bhutta, Zulfiqar; The Aga Khan University, Division of Women and Child Health; SickKids
**Primary Subject Heading**:	Paediatrics
Secondary Subject Heading:	Gastroenterology and hepatology, Global health, Infectious diseases, Paediatrics
Keywords:	Nutritional support < GASTROENTEROLOGY, Paediatric gastroenterology < GASTROENTEROLOGY, NUTRITION & DIETETICS, PUBLIC HEALTH, Paediatric infectious disease & immunisation < PAEDIATRICS, Paediatric gastroenterology < PAEDIATRICS

SCHOLARONE™
Manuscripts

A Protocol for a Systematic Review on Routine Use of Antibiotics for Infants Less Than Six Months of Age with Growth Failure/Faltering

Aamer Imdad, Fanny F Chen, Melissa Francois, Emily Tanner-Smith, Abigail Smith, Olivia J Tsistinas, Jai Das, Zulfiqar Ahmed Bhutta

Authors' information

Contact Author
Aamer Imdad, MBBS, MPH
Assistant Professor of Pediatrics
Division of Pediatric Gastroenterology, Hepatology and Nutrition
SUNY Upstate Medical University, Syracuse, NY
725 Irving Street, Syracuse, NY 13210
Email: imdada@upstate.edu
Phone: 315-464-8444
Fax: 315-464-8443

Fanny Chen
SUNY Upstate Medical University
ChenF@upstate.edu

Melissa Francois
SUNY Upstate Medical University
FrancoMe@upstate.edu

Emily Tanner-Smith
University of Oregon
etanners@uoregon.edu

For peer review only

For peer review only - http://bmjopen.bmj.com/site/about/guidelines.xhtml

Abigail Smith
SUNY Upstate Medical University
SmithAb@upstate.edu

Olivia Tsistinas
SUNY Upstate Medical University
TsistiJO@upstate.edu

Jai Das
Aga Khan University
Jai.das@aku.edu

Zulfiqar Ahmed Bhutta
Institute for Global Health & Development, The Aga Khan University
zulfiqar.bhutta@aku.edu

Key Words: Antibiotics, wasting, malnutrition, growth failure

Word Count: 2989

For peer review only

Abstract

Introduction: Antibiotics have been used as an adjunct in treating children with severe acute malnutrition 6 to 59 months of age; however, the data for infants less than six months is scarce. The World Health Organization (WHO) recently started guideline development for preventing and treating wasting, including growth failure/faltering in infants less than six months. This systematic review commissioned by WHO aims to synthesize evidence from current literature on the effectiveness of antibiotics for infants less than six months of age with growth failure/faltering.

Methods and Analysis

We will conduct a systematic review and meta-analysis for studies that assessed the effect of antibiotics in the treatment of infants with growth faltering. We will search multiple electronic databases. We will include randomized controls trials and non-randomized studies with a control arm. The study population is infants less than six months of age with growth failure. The intervention group will be infants who received no antibiotics or antibiotics other than recommended in 2013 guidelines by WHO to treat severe acute malnutrition in children. The comparison group will be infants who received antibiotics according to the 2013 guideline by WHO. We will consider the following outcomes: Mortality, Clinical deterioration, Antimicrobial resistance, Recovery from co-morbidity, Adverse events, markers of intestinal inflammation, markers of systemic inflammation, hospital-acquired infections, Non-response. We will use the meta-analysis to pool the studies where applicable. We will use the Grading of Recommendations Assessment, Development, and Evaluation (GRADE) approach to reporting the overall evidence quality for an outcome.

Ethics and Dissemination: This is a systematic review and will not involve contact with a human subject. The findings of this review will be published in a peer-review journal and will guide the WHO's recommendation for the use of antibiotics in infants less than six months of age with growth failure.

For peer review only

Article Summary: Strengths and Limitations

- We will search several electronic databases and include randomized and non-randomized studies.

-Two authors will screen the titles and extract the data from included studies duplication. We will assess the risk of bias for each outcome from each study and use the grading of recommendation, assessment, development and evaluation (GRADE) criteria to assess the overall quality of evidence

-We will conduct the meta-analysis if the data are available for more than one study for an outcome and there is clinical and methodological homogeneity in the included studies

-All the included studies may not report the data for all the outcomes mentioned in this review which will be a limitation of the study.

For peer review only

Main Manuscript

Introduction

The World Health Organization (WHO)and the United Nations Children's Fund (UNICEF) estimate that nearly 14 million children suffer from severe acute malnutrition (SAM) worldwide[1]. Infants less than six months of age are particularly vulnerable to the effects of inadequate nutrition. Higher mortality rates secondary to growth failure are seen in this age group compared to older infants and children[2,3]. Despite excess mortality risk and increasing prevalence of wasting in this population, limited studies exist to guide the management of young infants with growth failure and faltering [2-6]. Malnutrition not only increases the risk of severe infections in children and triples the risk of mortality from pneumonia, measles, or diarrhea, but also detrimentally affects their neurodevelopment [7-9]. Therefore, the current practice for children six months to 5 years of age with SAM is to prescribe routine antibiotics, such as 5 to 7 days of amoxicillin, when they are inpatients[9]. However, current recommendations state that the same general medical care should be used for infants with SAM who are less than six months of age as infants above six months of age, even though there is limited evidence to support this recommendation for infants less than six months of age[2]. Furthermore, even though antibiotics have been shown to be effective in children 6-59 months of age with SAM, this practice in infants has the potential to harm due to recently identified risks of antibiotic use in infancy, including the diminishment of infant gut microbiome[10], future development of obesity, allergic disorders[11], and autoimmune disorders[12]. The urgency in more targeted management guidelines is further underscored by the physiological differences in renal and gastrointestinal function in infants compared to older children[12]. The WHO recently started the process of guideline development for the prevention and treatment of wasting in children, including growth failure/faltering in infants less than six months. We aim to systematically review and synthesize evidence from current literature on the effectiveness of antibiotics for infants less than six months of age with growth failure/faltering.

Review question

In infants <6 months with growth failure/faltering, what are the effects of no routine antibiotics or different approaches (e.g., types of antibiotics, doses, etc.) compared to routine antibiotics following treatment protocols in the 2013 WHO guidelines on the outcomes of interest?

Methods

This systematic review will be conducted according to methods described in Cochrane Handbook[13] and reported using PRISMA guidelines 2020[14]. We registered the protocol on the PROSPERO website (ID CRD42021277073).

Inclusion Criteria

We will include randomized and non-randomized controlled trials. We will consider both individual and cluster randomized trials. We will also consider non-randomised trials and cohort studies with a controlled arm. Table 1 gives the definitions of the eligible study designs. We will exclude case-control studies, case reports, case series, and commentaries.

For peer review only - http://bmjopen.bmj.com/site/about/guidelines.xhtml

Population

The population of interest is infants under six months of age with growth failure/faltering. As there is no standard definition of growth failure/faltering, we will consider author definitions. We will include studies irrespective if they are done in the community setting or the hospital setting. We will consider studies that included infants infected with HIV. We will include studies with infants born low birth weight or preterm; however, we will exclude studies on infants admitted to the neonatal intensive care units. We will exclude studies that included infants with congenital anomalies only. If a study includes infants less than six months and greater than six months of age, we will consider this study if the disaggregated data are available for infants less than six months. If the disaggregated data are not available from such a study, we will consider the study if the average age of infants is at or below 6 months of age.

Intervention

We will include all antibiotic treatments given systemically, such as amoxicillin, Augmentin, cephalosporins, and macrolides. We will include studies irrespective of dosage, frequency, duration, or route of administration; however, topical application of antibiotics will not be considered. We will consider studies if the antibiotics were given empirically at the diagnosis of growth failure or faltering. We will also consider studies if the antibiotics were given in response to a suspected infection in infants with wasting. We will exclude studies where antibiotics were given for the prevention of wasting in otherwise healthy infants. We will also exclude studies where antibiotics were given for other reasons, such as suspected serious bacterial infections in otherwise healthy infants. We will allow co-intervention such as nutritional supplementation for nutritional rehabilitation only if similar in both groups.

Comparison

The comparison groups are routine antibiotics following treatment protocols detailed in the 2013 WHO guideline[2].

Outcomes

-Mortality (Dichotomous outcome)
-Clinical deterioration [Dichotomous outcome, defined by the development of any danger signs (obstructed breathing, respiratory distress, cyanosis, shock, severe anemia, convulsion, severe dehydration, profuse watery diarrhea, vomiting, and/or impaired consciousness)]
-Antimicrobial resistance (Dichotomous outcome, as defined by authors)
-Recovery from wasting- (Dichotomous outcome, as defined by authors)
-Recovery from co-morbidity, Pneumonia (Dichotomous outcome)
-Recovery from co-morbidity, diarrhea (Dichotomous outcome)
-Recovery from any co-morbidity (Dichotomous outcome)
-Adverse events-incidence of Diarrhea (Dichotomous outcome, > 3 loose stools/24 hours)
-Markers of intestinal inflammation-fecal calprotectin (continuous outcome)
-Markers of systemic inflammation-serum C reactive protein (continuous outcome)
-Hospital-acquired infections (Dichotomous outcome)

For peer review only

-Non-response (e.g., not achieving recovery within four months of initiating treatment) (dichotomous outcome)

We will consider all the outcomes at 1, 3, and 6 months and the longest follow-up. All the primary analyses will be done at the longest follow-up.

Balance of benefits and harms

We will stratify the outcomes by benefit and harm and provide a narrative summary of the balance of benefits and harms.

Literature search

We will conduct systematic electronic queries using key terms in multiple databases, including MEDLINE via PubMed, EMBASE, Web of Science, CINAHL, Scopus, LILACS, WHO Global Index Medicus, and BIOSIS Previews. There will be no search restrictions on outcomes, publication year, publication status, or publication language. The search strategies for different databases are available in Appendix 1. The references of formerly published reviews and recently published studies will be examined for potential inclusion. We will also use The Cochrane Central Register for Controlled Trials and ISRCTN registry to identify studies currently underway. We will also search the websites of pertinent international agencies such as the WHO (including WHO's Reproductive Health Library, electronic Library of Evidence of Nutrition Actions and Global database on the Implementation of Nutrition Action), UNICEF, Global Alliance for Improved Nutrition, International Food Policy Research Institute, International Initiative for Impact Evaluation, Nutrition International, World Bank, USAID and affiliates (e.g., FANTA, SPRING), and the World Food Programme. We will search the abstracts of the major conference, such as annual pediatric academic society meetings. Finally, we will use the citation tracking function of the included studies in PubMed to look for any eligible studies.

Data extraction and synthesis

Selection of studies and data extraction

We will use a three-phased approach to screen studies for eligibility. In the first phase, two authors will independently screen study titles and abstracts yielded from search results to identify potentially eligible ones. Studies selected during this initial phase will then go through a full-text review, which constitutes the second phase. In the final phase, studies that are determined to be eligible through the full-text review will undergo data extraction (figure 1). We will use the software Covidence in the screening process and create a form to store the data extracted in duplicate by two authors[15]. Two authors will screen the studies independently and compare their findings. The authors will resolve potential disagreements through discussions, and the senior author on the team may assist as needed.

We will extract characteristic data for each eligible study, including study region/country, study year, study type, intervention exposure (dose, duration, frequency), comparison, outcomes, population characteristics detailed in subgroup analysis, and risk of bias. We will also extract the information on feasibility, acceptability, equity, and resource use of the intervention and report this data in separate tables (Tables 2 and 3). We will extract the raw values for the number of events in the intervention and control group in case of dichotomous outcomes. To

For peer review only - http://bmjopen.bmj.com/site/about/guidelines.xhtml

avoid reviewer bias, we are deciding a priori the order of preference for extracting outcomes when data might be available in several formats. For randomized studies, we will prefer data that require the least manipulation by authors or inference by reviewers. We will give preference denominators in the following order for mortality data: number with the definite outcome known, number randomized, and child-years. For morbidity outcomes to which both survivors and non-survivors may have contributed data (for example, the incidence of diarrhea), we will give preference to child years/months/days, number with the definite outcome known, and number randomized.

For continuous outcomes from randomized trials, we will extract the raw values (for example, means and standard deviations) rather than calculated effect sizes (e.g., Cohen's d) and calculate the summary estimate ourselves. For denominators, we will preference the numbers with the definite outcome known and then number randomized. For non-randomized studies, we will prefer to extract the summary estimates (Mean difference or relative risk) adjusted for confounding variables.

If a study is only available in an abstract, we will contact the authors for full text. If the full text could not be obtained from any sources, we will include the abstract if sufficient details of study design and outcomes are available. We will try to find the protocol of each included study to assess the details of the methods. If the study protocol is not publicly available, we will contact the authors for the same. Suppose the results of the randomized trial are published in more than one report. In that case, we will consider all the publications related to that study as one study but extract the data from all relevant publications.

Assessment of risk of bias in included studies

We will evaluate the study risk of bias with the Cochrane risk of bias (ROB 2.0) and Cochrane ROBINS-I for randomized controlled trials and non-randomized studies of interventions, respectively[16,17]. Two authors will assess the risk of bias separately and compare their findings and reach a consensus. In the case of conflicts, a discussion will be held to resolve the conflict. A senior author will be consulted if a consensus could be reached between the two primary authors.

The risk of bias assessment according to ROB-2 and ROBINS-I is done for each outcome and not for a particular study[16,17]. This risk of bias assessment is different from earlier practice where a study was assessed for risk of bias, and the same assessment was applied to all the outcomes extracted from that study[13]. We will summarize the risk of bias for each domain (such as sequence generation, allocation concealment, etc.) and the overall risk of bias from the study for a particular outcome. The risk of bias will be summarized as high, low, or some concerns. We will present the risk of bias assessment in the form of graphs.

Figure 1: PRISMA diagram of data extraction process

Data Synthesis

We will report review findings both qualitatively and quantitatively. A narrative synthesis will be used to report the characteristics of all included studies and their results. A random-effects meta-analysis will be employed when at least two studies possess sufficient clinical and

For peer review only - http://bmjopen.bmj.com/site/about/guidelines.xhtml

methodological uniformity for synthesis. If quantitative synthesis is not appropriate, results will be described according to the standard guidelines of narrative synthesis[18]. The software RevMan will be utilized for statistical analysis[19]. Dichotomous outcomes will be assessed using relative risk (RR) effect sizes and presented with 95% confidence intervals (CI). The continuous outcomes will be combined to obtain a standardized mean difference and reported with a 95 % CI. We will present the meta-analysis results in the form of forest plots and examine their statistical heterogeneity and inconsistency.

We will include the data from randomized controlled trials (RCT) on an intention to treat analysis basis. If an RCT does not report an intention to treat analysis, we will generate our own intention to treat analysis by using the raw values extracted from the trial. Suppose an RCT does not report the raw values but reports the summary estimate, such as relative risk. In that case, we will include the summary estimate using the generic inverse variance weighting method of meta-analysis. For observational studies, we will prefer to use a summary estimate that was adjusted for confounding factors. If a non-randomized study reports an odd ratio as the summary estimate, we will calculate the relative risk if the raw data are available. If the raw data are not available to calculate a relative risk, we will pool the odd ratio if the large study sample size. We will do the sensitivity analysis by excluding such studies to see if it has any detrimental effect on the final summary estimate of the pooled analysis.

We plan to pool the data separately from observational studies and randomized studies. Suppose the number of studies is small (less than 10). In that case, we may pool both the study designs in the same analysis but do a sensitivity analysis by excluding the observational studies.

We will consider the following two comparisons.

1) No routine antibiotics compared to routine antibiotics following treatment protocols in the 2013 WHO guidelines

2) Different approaches to the use of antibiotics (e.g., types of antibiotics, doses, etc.) compared to the routine antibiotics following treatment protocols in the 2013 WHO guidelines

Studies with missing data

We will record missing data during the data extraction process. We will use imputations of missing data over recording the data as missing. If data are missing for some cases or dropouts are not reported, we will contact the study authors requesting the complete data. If a study does imputations for the missing data, we will use the imputed data. If a study does not report the raw values, we will extract the summary estimates such as relative risk or risk ratio. If a study does not report mean but median, we will convert the median to mean based on the Cochrane Handbook of Systematic Reviews methods[13]. Suppose the SD is not available for a mean, and the value cannot be calculated from available data such as standard error, confidence interval, or the p-value. In that case, we will contact the authors for additional information. If the SD is not available from any source for a mean, we will use the SD from a

For peer review only - http://bmjopen.bmj.com/site/about/guidelines.xhtml

similar study. If the data are presented only in graphs, we will extract the information to the best possible approximation.

Assessment of heterogeneity

We will analyze statistical heterogeneity in the pooled data using Tau^2, χ^2, and I^2 statistics. We will also assess statistical heterogeneity through visual inspection of forest plots, utilizing the χ^2 test (assessing the p-value) and calculating the Tau^2 and I^2 statistics. We will consider it significant statistical heterogeneity when the p-value is less than 0.1, the I2 value exceeds 50%, and the inspection of forest plots shows substantial variability in the effect of the intervention. Finally, we will perform subgroup analysis to identify reasons for eligible statistical heterogeneity.

Assessment of reporting bias

We will assess publication bias of small studies using funnel plots and regression tests for funnel plot asymmetry when the meta-analysis includes at least ten studies.

Subgroup analysis and investigation of heterogeneity

- By different types/definitions of growth failure/faltering
- Age at presentation [newborn (0-28 days), 1-3 months, 4-6 months]
- Gestational age: Preterm birth (< 37 weeks) vs. full-term birth (> 37 weeks)
- Birth weight: Low birth weight (<2500 g) vs. Normal birth weight (> 2500 g)
- HIV exposure: Studies with Participants exposed to HIV VS. Studies with no HIV exposure
- Presentation: Participants with Oedema vs. Patricians with no oedema
- Co-morbidities: With or without co-morbidities
- Nutrition: Babies Breastfeeding or non-breastfed babies
- Location of the treatment: inpatient or outpatient/community
- Dose of antibiotics
- Duration of antibiotics: 7 days vs. > 7 days
- Type of antibiotics

The subgroup analyses above will be based on individual-level data. We will try to extract the information from individual studies as available. If the information is not available from the published studies, we will write to the authors to get the required data.

Sensitivity analysis

We will complete sensitivity analysis by removing studies with a high risk of bias. We will compare results from random versus fixed-effect meta-analysis models.

Rating of overall quality of evidence

The Grading of Recommendations Assessment, Development, and Evaluation (GRADE) approach will be used to evaluate the overall certainty of evidence using the software GRADEpro[20]. The GRADE approach is a comprehensive framework used to assess the overall certainty of the evidence for an outcome utilizing study characteristics such as study design,

For peer review only - http://bmjopen.bmj.com/site/about/guidelines.xhtml

inconsistency, indirectness of evidence, risk of bias, publication bias, and imprecision estimates. We will include the GRADE assessment results in a GRADE evidence profile that contains certainty ratings, including very low, low, moderate, or high based on the evidence across studies for primary outcomes (table 4).

Amendments

We will conduct literature searches, screening of titles, selection of studies, data extraction, and analysis according to the plan aforementioned in the protocol. We will report any additional analysis performed in the methods section if such occurs.

Patient and public involvement

No patient or public involvement was considered in the preparation of this protocol.

Consent for publication

The authors give consent for the publication of the review.

Availability of data and materials

We will provide data available for review by the editors and peer reviewers and provide raw data for the general public on request.

Ethics and dissemination

This review is intended as background work for WHO's guide to the therapeutic use of antibiotics in malnourished infants under six months. Considering this is a systematic review, there will be no direct contact with the patients included in this study. The findings stemming from this systematic review will be distributed to relevant populations, including patients, caregivers, and healthcare workers.

Author's contribution

AI and ZAB conceptualized the study. FC and AI wrote the first draft of the manuscript. MF contributed to method. ETS contributed to methods. AS and OJT designed the search strategy. JD, ZAB provided the supervision as experts and contributed to methods and manuscript writing.

Competing interests

No, there are no competing interests for any author

Funding

This work is funded by World Health Organization grant no 202725572. WHO also provided technical support for this work.

Acknowledgements

We are very thankful to Allison Daniel, Jaden BENDABENDA, Kirrily De POLNAY and Zita WEISE PRINZO for their input to improve this protocol.

For peer review only - http://bmjopen.bmj.com/site/about/guidelines.xhtml

For peer review only

Table 1: Suggested study designs for inclusion in the review

Suggested terms	Definition	Notes
Randomised controlled trial (RCT) OR randomised trial	An experimental study in which people are allocated to different interventions using methods that are random.	We will consider both individual and cluster randomized trials. We also consider the factorial design trials
Non-randomised controlled trial (NRCT) OR non-randomised trial	An experimental study in which people are allocated to different interventions using methods that are not random.	We will use consistent terminology and avoid using the term quasi-experimental as it might have different meanings in different settings. We will use the ROBINS-1 for risk of bias assessment for this study design
Controlled before-after study (CBA)	A study in which observations are made before and after the implementation of an intervention, both in a group that receives the intervention and in a control group that does not.	We will require two minimum criteria for the inclusion of CBAs.<br>-Data Collection: We will include CBAs if the data for the intervention and control groups were collected prospectively in the same time frame<br>-Choice of Control: We will include CBAs that include a control at a second site to avoid contamination of the intervention to the control group if the settings and populations are the same for the intervention and control groups.

This table is modified from:
https://epoc.cochrane.org/sites/epoc.cochrane.org/files/public/uploads/EPOC%20Study%20Designs%20About.pdf

Table 2: Sample table to report the qualitative data from the included studies

Study	Study population	Intervention studied	Feasibility of intervention	Acceptability of intervention	Considerations for Equity

Table 3: Sample table for a description of qualitative data on resource use

**Study**	**Intervention studies**	**Resources used for production**	**Resources used for administration**

For peer review only - http://bmjopen.bmj.com/site/about/guidelines.xhtml

			**of the intervention**

Table 4: Description of methods for grading of overall evidence

<table>
<tr><td>Study Design</td><td>Quality of Evidence</td><td>Lower Certainty Score if</td><td>Higher certainty score if</td></tr>
<tr><td rowspan="2">Randomized trial</td><td>High</td><td rowspan="4">Risk of Bias
-1 Serious
-2 Very serious

Inconsistency
-1 Serious
-2 Very serious

Indirectness
-1 Serious
-2 Very serious

Imprecision
-1 Serious
-2 Very serious

Publication Bias
-1 Likely
-2 Very likely</td><td rowspan="4">Large effect
+1 Large
+2 Very large

Dose-response
+1 evidence of a gradient

All plausible confounding would:
+1 Reduce a demonstrated effect

+1 Suggest a spurious effect when results show no effect</td></tr>
<tr><td>Moderate</td></tr>
<tr><td rowspan="2">Observational study</td><td>Low</td></tr>
<tr><td>Very Low</td></tr>
</table>

* In the GRADE approach, randomized controlled trials (RCTs) start as high-quality evidence and observational studies as low-quality evidence supporting estimates of intervention effects. Five factors may lead to rating down the quality of evidence, and three factors may lead to rating up. Ultimately, the quality of evidence for each outcome falls into one of four categories, from high to very low. GRADE is "outcome centric": rating is made for each outcome, and quality may differ indeed, is likely to differ from one outcome to another within a single study and across a body of evidence. [21]

References

1. United Nations Children's Fund (UNICEF), World Health Organization, International Bank for Reconstruction and Development/The World Bank. Levels and trends in child malnutrition: Key Findings of the 2020 Edition of the Joint Child Malnutrition Estimates. Geneva: World Health Organization. Licence: CC BY-NC-SA 3.0 IGO. 2020.
2. WHO. Guideline: Updates on the management of severe acute malnutrition in infants and children. Geneva: World Health Organization. 2013.
3. Kerac M, Blencowe H, Grijalva-Eternod C, et al. Prevalence of wasting among under 6-month-old infants in developing countries and implications of new case definitions using WHO growth standards: a secondary data analysis. Arch Dis Child 2011;96(11):1008-13. DOI: 10.1136/adc.2010.191882.
4. Nasrin D, Blackwelder WC, Sommerfelt H, et al. Pathogens Associated With Linear Growth Faltering in Children With Diarrhea and Impact of Antibiotic Treatment: The

For peer review only

For peer review only - http://bmjopen.bmj.com/site/about/guidelines.xhtml

Global Enteric Multicenter Study. J Infect Dis 2021;224(12 Suppl 2):S848-S855. DOI: 10.1093/infdis/jiab434.
5. Brander RL, Pavlinac PB, Walson JL, et al. Determinants of linear growth faltering among children with moderate-to-severe diarrhea in the Global Enteric Multicenter Study. BMC Med 2019;17(1):214. DOI: 10.1186/s12916-019-1441-3.
6. Patwari AK, Kumar S, Beard J. Undernutrition among infants less than 6 months of age: an underestimated public health problem in India. Matern Child Nutr 2015;11(1):119-26. DOI: 10.1111/mcn.12030.
7. Prado EL, Larson LM, Cox K, Bettencourt K, Kubes JN, Shankar AH. Do effects of early life interventions on linear growth correspond to effects on neurobehavioural development? A systematic review and meta-analysis. Lancet Glob Health 2019;7(10):e1398-e1413. DOI: 10.1016/S2214-109X(19)30361-4.
8. Shekhar S, Petersen FC. The Dark Side of Antibiotics: Adverse Effects on the Infant Immune Defense Against Infection. Front Pediatr 2020;8:544460. DOI: 10.3389/fped.2020.544460.
9. Alcoba G, Kerac M, Breysse S, et al. Do children with uncomplicated severe acute malnutrition need antibiotics? A systematic review and meta-analysis. PLoS One 2013;8(1):e53184. DOI: 10.1371/journal.pone.0053184.
10. Stam J, van Stuijvenberg M, Gruber C, et al. Antibiotic use in infants in the first year of life in five European countries. Acta Paediatr 2012;101(9):929-34. DOI: 10.1111/j.1651-2227.2012.02728.x.
11. Droste JH, Wieringa MH, Weyler JJ, Nelen VJ, Vermeire PA, Van Bever HP. Does the use of antibiotics in early childhood increase the risk of asthma and allergic disease? Clin Exp Allergy 2000;30(11):1547-53. DOI: 10.1046/j.1365-2222.2000.00939.x.
12. Trasande L, Blustein J, Liu M, Corwin E, Cox LM, Blaser MJ. Infant antibiotic exposures and early-life body mass. Int J Obes (Lond) 2013;37(1):16-23. DOI: 10.1038/ijo.2012.132.
13. Higgins JPT TJ, Chandler J, Cumpston M, Li T, Page MJ, Welch VA (editors). Cochrane Handbook for Systematic Reviews of Interventions version 6.2 Available from www.training.cochrane.org/handbook. 2021.
14. Page MJ, McKenzie JE, Bossuyt PM, et al. The PRISMA 2020 statement: an updated guideline for reporting systematic reviews. BMJ 2021;372:n71. DOI: 10.1136/bmj.n71.
15. Covidence systematic review software. veritas health innovation. Melbourne, Australia: www.covidence.org; 2021.
16. Sterne JAC, Savovic J, Page MJ, et al. RoB 2: a revised tool for assessing risk of bias in randomised trials. BMJ 2019;366:l4898. DOI: 10.1136/bmj.l4898.
17. Sterne JA, Hernan MA, Reeves BC, et al. ROBINS-I: a tool for assessing risk of bias in non-randomised studies of interventions. BMJ 2016;355:i4919. DOI: 10.1136/bmj.i4919.
18. Campbell M, McKenzie JE, Sowden A, et al. Synthesis without meta-analysis (SWiM) in systematic reviews: reporting guideline. BMJ 2020;368:l6890. DOI: 10.1136/bmj.l6890.
19. Review Manager (RevMan). 5.3 ed. Copenhagen: The Nordic Cochrane Center TCC; 2014.
20. GRADEpro GDT: GRADEpro guideline development tool. McMaster University: Evidence Prime, Inc; 2020.

For peer review only

21. Guyatt G, Oxman AD, Akl EA, et al. GRADE guidelines: 1. Introduction-GRADE evidence profiles and summary of findings tables. J Clin Epidemiol 2011;64(4):383-94. DOI: 10.1016/j.jclinepi.2010.04.026.

For peer review only

For peer review only - http://bmjopen.bmj.com/site/about/guidelines.xhtml

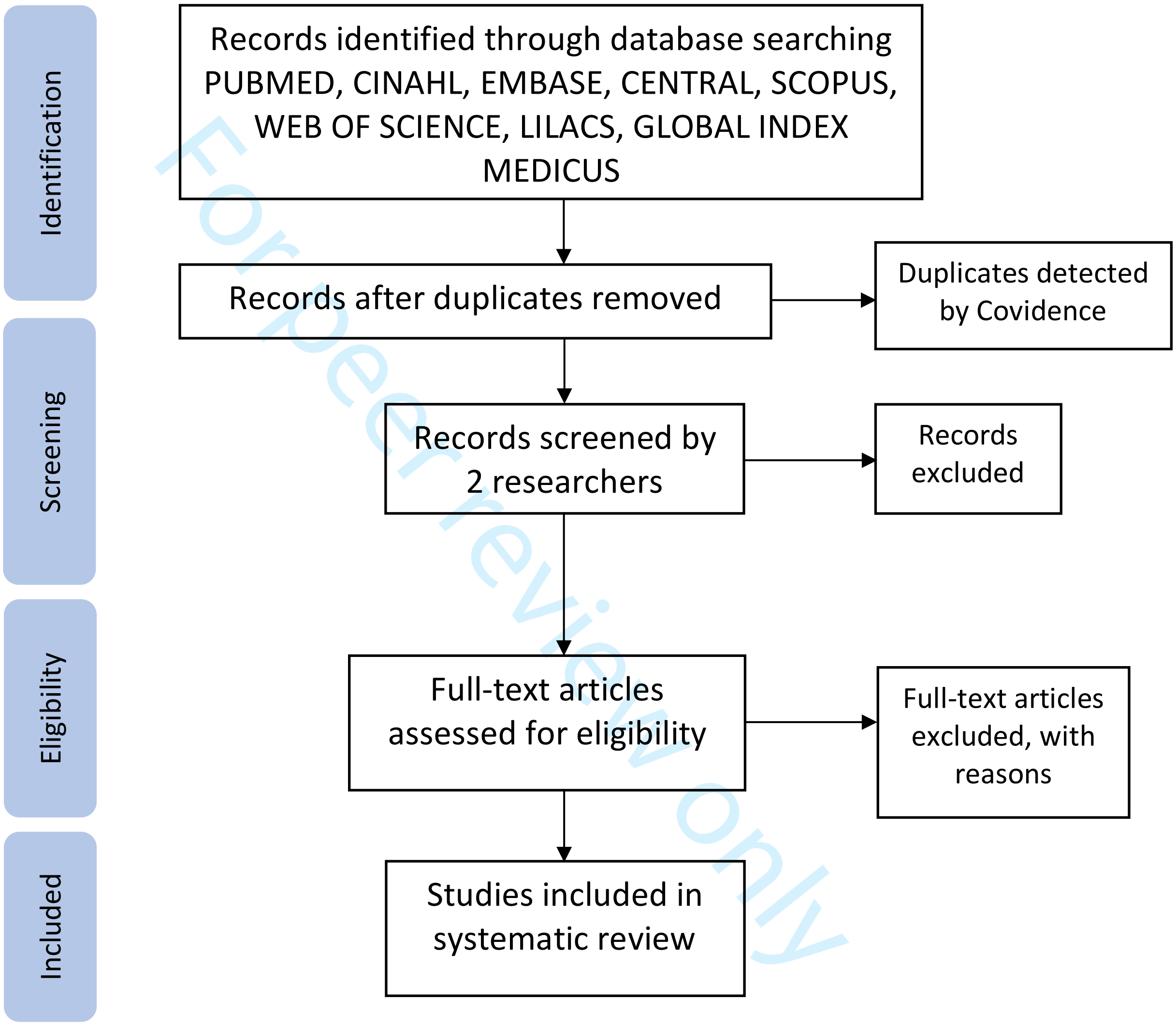

For peer review only

Appendix 1

Search Strategies for Growth Failure AND Antibiotics

PubMed

(("Infant"[MeSH Terms] OR "Child"[MeSH Terms:noexp] OR "Premature Birth"[MeSH Terms] OR ("infant*"[Title/Abstract] OR "infancy"[Title/Abstract] OR "baby"[Title/Abstract] OR "babies"[Title/Abstract] OR "neonat*"[Title/Abstract] OR "neo nat*"[Title/Abstract] OR "newborn*"[Title/Abstract] OR "new born*"[Title/Abstract] OR "newly born*"[Title/Abstract] OR "preterm"[Title/Abstract] OR "preterms"[Title/Abstract] OR "pre term"[Title/Abstract] OR "pre terms"[Title/Abstract] OR "lbw"[Title/Abstract] OR "vlbw"[Title/Abstract] OR "elbw"[Title/Abstract] OR "child*"[Title/Abstract] OR "youth"[Title/Abstract] OR "juvenile*"[Title/Abstract]) OR ("premature*"[Title/Abstract] AND ("birth*"[Title/Abstract] OR "born"[Title/Abstract] OR "deliver*"[Title/Abstract])) OR ("low"[Title/Abstract] AND ("birthweight*"[Title/Abstract] OR "birth weight*"[Title/Abstract]))) AND ("Malnutrition"[MeSH Terms] OR "Failure to Thrive"[MeSH Terms] OR "Growth Disorders"[MeSH Terms:noexp] OR "Severe Acute Malnutrition"[MeSH Terms] OR "Wasting Syndrome"[MeSH Terms:noexp] OR "Infant Nutrition Disorders"[MeSH Terms:noexp] OR ("growth failure*"[Title/Abstract] OR "growth falter*"[Title/Abstract] OR "slow growth"[Title/Abstract] OR "slowed growth"[Title/Abstract] OR "Malnutrition"[Title/Abstract] OR "malnourish*"[Title/Abstract] OR "Severe Acute Malnutrition"[Title/Abstract] OR "SAM"[Title/Abstract] OR "wasting"[Title/Abstract] OR "wasted"[Title/Abstract] OR "growth restrict*"[Title/Abstract] OR "growth retard*"[Title/Abstract] OR "Failure to Thrive"[Title/Abstract] OR "FTT"[Title/Abstract] OR "growth disorder*"[Title/Abstract] OR "growth arrest*"[Title/Abstract] OR "growth deficien*"[Title/Abstract] OR "growth disturb*"[Title/Abstract])) AND ("Anti-Bacterial Agents"[Pharmacological Action] OR "Anti-Bacterial Agents"[MeSH Terms] OR ("antibiotic*"[Title/Abstract] OR "anti bacterial agent*"[Title/Abstract] OR "antibacterial*"[Title/Abstract] OR "anti bacterial*"[Title/Abstract] OR "bactericid*"[Title/Abstract] OR "bacteriocid*"[Title/Abstract] OR "anti mycobacterial*"[Title/Abstract] OR "antimycobacterial*"[Title/Abstract]))) NOT ("Animals"[MeSH Terms] NOT ("Animals"[MeSH Terms] AND "Humans"[MeSH Terms]))

CINAHL

S1 (MH "Infant+")
S2 (MH "Child")
S3 (MH "Childbirth, Premature")
S4 TI (infant* OR infancy OR baby OR babies OR neonat* OR "neo nat*" OR newborn* OR "new

born*" OR "newly born*" OR preterm OR preterms OR "pre term" OR "pre terms" OR lbw OR vlbw
OR elbw OR child* OR youth OR juvenile*)
S5 AB (infant* OR infancy OR baby OR babies OR neonat* OR "neo nat*" OR newborn* OR "new born*" OR "newly born*" OR preterm OR preterms OR "pre term" OR "pre terms" OR lbw OR vlbw
OR elbw OR child* OR youth OR juvenile*)
S6 TI (premature* N2 (birth* OR born OR deliver*))
S7 AB (premature* N2 (birth* OR born OR deliver*))
S8 TI (low N2 (birthweight* OR "birth weight*"))
S9 AB (low N2 (birthweight* OR "birth weight*"))
S10 S1 OR S2 OR S3 OR S4 OR S5 OR S6 OR S7 OR S8 OR S9

S11 (MH "Malnutrition")
S12 (MH "Failure to Thrive")
S13 (MH "Growth Disorders")
S14 (MH "Wasting Syndrome")
S15 (MH “Infant Nutrition Disorders”)
S16 TI ((grow*) N2 (fail* OR falter* OR slow* OR restrict* OR retard* OR deficien* OR arrest* OR disturb* OR disorder*))
S17 AB ((grow*) N2 (fail* OR falter* OR slow* OR restrict* OR retard* OR deficien* OR arrest* OR disturb* OR disorder*))
S18 TI ((fail*) W2 (thrive))
S19 AB ((fail*) W2 (thrive))
S20 TI (Malnutrition OR malnourish* OR "Severe Acute Malnutrition" OR SAM OR wasting OR wasted OR FTT)
S21 AB (Malnutrition OR malnourish* OR "Severe Acute Malnutrition" OR SAM OR wasting OR wasted OR FTT)
S22 S11 OR S12 OR S13 OR S14 OR S15 OR S16 OR S17 OR S18 OR S19 OR S20 OR S21
S23 (MH "Antibiotics+")
S24 TI (antibiotic* OR "anti bacterial*" OR "antibacterial*" OR bactericid*OR bacteriocid* OR "anti
mycobacterial*" OR "antimycobacterial*")
S25 AB (antibiotic* OR "anti bacterial*" OR "antibacterial*" OR bactericid* OR bacteriocid* OR
"anti mycobacterial*" OR "antimycobacterial*")
S26 S23 OR S24 OR S25
S27 (MH "Animals") NOT ((MH "Animals") AND (MH "Human"))
S28 S10 AND S22 AND S26
S29 S28 NOT S27
S30 S28 NOT S27 Exclude Medline Records

Embase

#1'infant'/exp OR 'newborn'/exp OR 'prematurity'/exp OR 'low birth weight'/exp OR 'very low birth
weight'/exp OR 'extremely low birth weight'/exp OR 'juvenile'/de OR 'child'/de
#2 infant*:ti,ab OR infancy:ti,ab OR baby:ti,ab OR babies:ti,ab OR neonat*:ti,ab OR 'neo nat*':ti,ab OR newborn*:ti,ab OR 'new born*':ti,ab OR 'newly born*':ti,ab OR preterm:ti,ab OR preterms:ti,ab
OR 'pre term':ti,ab OR 'pre terms':ti,ab OR lbw:ti,ab OR vlbw:ti,ab OR elbw:ti,ab OR child*:ti,ab OR
youth:ti,ab OR juvenile*:ti,ab
#3 (premature* NEAR/2 (birth* OR born OR deliver*)):ti,ab

For peer review only

#4 (low NEAR/2 (birthweight* OR 'birth weight*')):ti,ab
#5 #1 OR #2 OR #3 OR #4

#6 'malnutrition'/exp OR 'failure to thrive'/exp OR 'growth disorder'/de OR 'wasting syndrome'/de OR 'growth retardation'/de
#7(grow* NEAR/2 (fail* OR falter* OR slow* OR restrict* OR retard* OR deficien* OR arrest* OR disturb* OR disorder*)):ti,ab
#8 (fail* NEXT/2 thrive):ti,ab
#9 malnutrition:ti,ab OR malnourish*:ti,ab OR 'severe acute malnutrition':ti,ab OR sam:ti,ab OR wasting:ti,ab OR wasted:ti,ab OR ftt:ti,ab
#10 #6 OR #7 OR #8 OR #9
#11'antibiotic agent'/exp OR 'bactericide'/de OR 'antimycobacterial agent'/de
#12 antibiotic*:ti,ab OR 'anti bacterial*':ti,ab OR 'antibacterial*':ti,ab OR bactericid*:ti,ab OR bacteriocid*:ti,ab OR antimycobacterial*:ti,ab OR 'anti mycobacterial*':ti,ab
#13 #11 OR #12
#14 #5 AND #10 AND #13
#15 #14 NOT ('animals'/exp NOT 'humans'/exp)
#16 #15 NOT [medline]/lim

CENTRAL

#1 MeSH descriptor: [Infant] explode all trees
#2 MeSH descriptor: [Child] this term only
#3 MeSH descriptor: [Premature Birth] explode all trees
#4 infant*:ti,ab OR infancy:ti,ab OR baby:ti,ab OR babies:ti,ab OR neonat*:ti,ab OR newborn*:ti,ab OR preterm:ti,ab OR preterms:ti,ab OR lbw:ti,ab OR vlbw:ti,ab OR elbw:ti,ab OR child*:ti,ab OR youth:ti,ab OR juvenile*:ti,ab
#5 (neo NEXT nat* OR new NEXT born* OR newly NEXT born* OR pre NEXT term OR pre NEXT terms):ti,ab
#6 (premature* NEAR/2 (birth* OR born OR deliver*)):ti,ab
#7 (low NEAR/2 (birthweight* OR birth NEXT weight*)):ti,ab
#8 #1 OR #2 OR #3 OR #4 OR #5 OR #6 OR #7
#9 MeSH descriptor: [Malnutrition] explode all trees
#10 MeSH descriptor: [Failure to Thrive] explode all trees
#11 MeSH descriptor: [Growth Disorders] this term only
#12 MeSH descriptor: [Severe Acute Malnutrition] explode all trees
#13 MeSH descriptor: [Wasting Syndrome] this term only
#14 MeSH descriptor: [Infant Nutrition Disorders] this term only
#15 ((grow*) NEAR/2 (fail* OR falter* OR slow* OR restrict* OR retard* OR deficien* OR arrest* OR disturb* OR disorder*)):ti,ab

#16 ((fail*) NEXT/2 (thrive)):ti,ab
#17 (severe NEXT acute NEXT malnutrition):ti,ab
#18 malnutrition:ti,ab OR malnourish*:ti,ab OR sam:ti,ab OR wasting:ti,ab OR wasted:ti,ab OR ftt:ti,ab
#19 #9 OR #10 OR #11 OR #12 OR #13 OR #14 OR #15 OR #16 OR #17 OR #18
#20 MeSH descriptor: [Anti-Bacterial Agents] explode all trees
#21 antibiotic*:ti,ab OR antibacterial*:ti,ab OR bactericid*:ti,ab OR bacteriocid*:ti,ab OR antimycobacterial*:ti,ab
#22 (anti NEXT bacterial*):ti,ab
#23 (anti NEXT mycobacterial*):ti,ab
#24 #20 OR #21 OR #22 OR #23
#25 #8 AND #19 AND #24
#26 MeSH descriptor: [Animals] explode all trees

For peer review only

#27 MeSH descriptor: [Humans] explode all trees
#28 (#26 NOT (#26 AND #27))
#29 #25 NOT #28
#30 "accession number" NEAR pubmed
#31 #29 NOT #30

Scopus

((TITLE-ABS (infant* OR infancy OR baby OR babies OR neonat* OR "neo nat*" OR
newborn* OR "new born*" OR "newly born*" OR preterm OR preterms OR "pre
term" OR
"pre terms" OR lbw OR vlbw OR elbw OR child* OR youth OR juvenile*)) OR (TITLE-ABS
(low W/2 (birthweight* OR "birth weight*"))) OR (TITLE-ABS (premature* W/2 (birth* OR
born OR deliver*)))) AND ((TITLE-ABS ((grow*) W/2 (fail* OR falter* OR slow* OR
restrict* OR retard* OR deficien* OR arrest* OR disturb* OR disorder*))) OR (TITLE-ABS (
(fail*) PRE/2 (thrive))) OR (TITLE-ABS (malnutrition OR malnourish* OR "Severe Acute
Malnutrition" OR sam OR wasting OR wasted OR ftt))) AND (TITLE-ABS (antibiotic* OR
"anti bacterial*" OR "antibacterial*" OR bactericid* OR bacteriocid* OR "anti
mycobacterial*"
OR "antimycobacterial*")) AND NOT INDEX (medline)

Web of Science

#1 TI=(infant* OR infancy OR baby OR babies OR neonat* OR "neo nat*" OR newborn* OR
"new born*" OR "newly born*" OR preterm OR preterms OR "pre term" OR
"pre terms" OR
lbw OR vlbw OR elbw OR child* OR youth OR juvenile*)
#2 AB=(infant* OR infancy OR baby OR babies OR neonat* OR "neo nat*" OR newborn* OR
"new born*" OR "newly born*" OR preterm OR preterms OR "pre term" OR
"pre terms" OR
lbw OR vlbw OR elbw OR child* OR youth OR juvenile*)
#3 TI=(premature* NEAR/2 (birth* OR born OR deliver*))

#4 AB=(premature* NEAR/2 (birth* OR born OR deliver*))
#5 TI=(low NEAR/2 (birthweight* OR "birth weight*";))
#6 AB=(low NEAR/2 (birthweight* OR "birth weight*";))
#7 #6 OR #5 OR #4 OR #3 OR #2 OR #1
#8 TI=((grow*) NEAR/2 (fail* OR falter* OR slow* OR restrict* OR retard* OR deficien* OR arrest*
OR disturb* OR disorder*))
#9 AB=((grow*) NEAR/2 (fail* OR falter* OR slow* OR restrict* OR retard* OR deficien* OR arrest*
OR disturb* OR disorder*))
#10 TI=((fail*) NEAR/2 (thrive))
#11 AB=((fail*) NEAR/2 (thrive))
#12 TI =(Malnutrition OR malnourish* OR "Severe Acute Malnutrition" OR SAM OR wasting OR
wasted OR FTT)
#13 AB=(Malnutrition OR malnourish* OR "Severe Acute Malnutrition" OR SAM OR wasting OR
wasted OR FTT)
#14 #13 OR #12 OR #11 OR #10 OR #9 OR #8
#15 TI=(antibiotic* OR "anti bacterial*" OR "antibacterial*" OR bactericid* OR bacteriocid*
OR "anti
mycobacterial*" OR "antimycobacterial*";)
#16 AB=(antibiotic* OR "anti bacterial*" OR "antibacterial*" OR bactericid* OR
bacteriocid* OR
"anti mycobacterial*" OR "antimycobacterial*";)
#17 #16 OR #15
#18 #17 AND #14 AND #7

LILACS

For peer review only

(mh:(infant)) OR ((mh:(child))) OR ((mh:("Premature Birth"))) OR ((ti:((premature* AND (birth* OR born OR deliver*))))) OR ((ab:((premature* AND (birth* OR born OR deliver*))))) OR ((ti:((low AND (birthweight* OR "birth weight*"))))) OR ((ab:((low AND (birthweight* OR "birth weight*"))))) OR
((ti:(infant* OR infancy OR baby OR babies OR neonat* OR "neo nat*" OR newborn* OR "new born*" OR "newly born*" OR preterm OR preterms OR "pre term" OR "pre terms" OR lbw OR vlbw
OR elbw OR child* OR youth OR juvenile*))) OR ((ab:(infant* OR infancy OR baby OR babies OR neonat* OR "neo nat*" OR newborn* OR "new born*" OR "newly born*" OR preterm OR preterms
OR "pre term" OR "pre terms" OR lbw OR vlbw OR elbw OR child* OR youth OR juvenile*)))
AND
((mh:(malnutrition)) OR ((mh:("Failure to Thrive"))) OR ((mh:("Growth Disorders"))) OR ((mh:("Severe Acute Malnutrition"))) OR ((mh:("Wasting Syndrome"))) OR ((mh:("Infant Nutrition
Disorders"))) OR ((ti:("growth failure*" OR "growth falter*" OR "slow growth" OR "slowed growth"
OR malnutrition OR malnourish* OR "Severe Acute Malnutrition" OR sam OR wasting OR wasted
OR "growth restrict*" OR "growth retard*" OR "Failure to Thrive" OR ftt OR "growth disorder*" OR
"growth arrest*" OR "growth deficien*" OR "growth disturb*"))) OR ((ab:("growth failure*" OR
"growth falter*" OR "slow growth" OR "slowed growth" OR malnutrition OR malnourish* OR
"Severe Acute Malnutrition" OR sam OR wasting OR wasted OR "growth restrict*" OR "growth
retard*" OR "Failure to Thrive" OR ftt OR "growth disorder*" OR "growth arrest*" OR "growth
deficien*" OR "growth disturb*")))) AND ((mh:("Anti-Bacterial Agents";)) OR ((ti:(antibiotic* OR
"anti bacterial agent*" OR antibacterial* OR "anti bacterial*" OR "bactericid*" OR "bacteriocid*"

OR "anti mycobacterial*" OR antimycobacterial*))) OR ((ab:(antibiotic* OR "anti bacterial agent*"
OR antibacterial* OR "anti bacterial*" OR "bactericid*" OR "bacteriocid*" OR "anti mycobacterial*"
OR antimycobacterial*)))) AND NOT ((mh:(animals)) AND NOT ((mh:(animals)) AND ((mh:(humans)))))
Global Index Medicus
(mh:(infant)) OR ((mh:(child))) OR ((mh:("Premature Birth"))) OR ((ti:((premature* AND (birth* OR born OR deliver*))))) OR ((ab:((premature* AND (birth* OR born OR deliver*))))) OR ((ti:((low AND (birthweight* OR "birth weight*"))))) OR ((ab:((low AND (birthweight* OR "birth weight*"))))) OR
((ti:(infant* OR infancy OR baby OR babies OR neonat* OR "neo nat*" OR newborn* OR "new born*" OR "newly born*" OR preterm OR preterms OR "pre term" OR "pre terms" OR lbw OR vlbw
OR elbw OR child* OR youth OR juvenile*))) OR ((ab:(infant* OR infancy OR baby OR babies OR neonat* OR "neo nat*" OR newborn* OR "new born*" OR "newly born*" OR preterm OR preterms
OR "pre term" OR "pre terms" OR lbw OR vlbw OR elbw OR child* OR youth OR juvenile*)))
AND
((mh:(malnutrition)) OR ((mh:("Failure to Thrive"))) OR ((mh:("Growth Disorders"))) OR

For peer review only

((mh:("Severe Acute Malnutrition"))) OR ((mh:("Wasting Syndrome"))) OR ((mh:("Infant Nutrition Disorders"))) OR ((ti:("growth failure*" OR "growth falter*" OR "slow growth" OR "slowed growth" OR malnutrition OR malnourish* OR "Severe Acute Malnutrition" OR sam OR wasting OR wasted OR "growth restrict*" OR "growth retard*" OR "Failure to Thrive" OR ftt OR "growth disorder*" OR "growth arrest*" OR "growth deficien*" OR "growth disturb*"))) OR ((ab:("growth failure*" OR "growth falter*" OR "slow growth" OR "slowed growth" OR malnutrition OR malnourish* OR "Severe Acute Malnutrition" OR sam OR wasting OR wasted OR "growth restrict*" OR "growth retard*" OR "Failure to Thrive" OR ftt OR "growth disorder*" OR "growth arrest*" OR "growth deficien*" OR "growth disturb*")))) AND ((mh:("Anti-Bacterial Agents")) OR ((ti:(antibiotic* OR "anti bacterial agent*" OR antibacterial* OR "anti bacterial*" OR "bactericid*" OR "bacteriocid*" OR "anti mycobacterial*" OR antimycobacterial*))) OR ((ab:(antibiotic* OR "anti bacterial agent*" OR antibacterial* OR "anti bacterial*" OR "bactericid*" OR "bacteriocid*" OR "anti mycobacterial*" OR antimycobacterial*)))) AND NOT ((mh:(animals)) AND NOT ((mh:(animals)) AND ((mh:(humans))))) AND (collection_gim:("IMSEAR" OR "WPRIM" OR "IMEMR" OR "AIM"))

For peer review only

Routine antibiotics for infants less than six months of age with growth failure/faltering. A protocol for the systematic review

Aamer Imdad, Fanny F Chen, Melissa Francois, Emily Tanner-Smith, Abigail Smith, Olivia J Tsistinas, Jai Das, Zulfiqar Ahmed Bhutta

PRISMA-P (Preferred Reporting Items for Systematic review and Meta-Analysis Protocols) 2015 checklist: recommended items to address in a systematic review protocol*

Section and topic	Item No	Checklist item	This manuscript
**ADMINISTRATIVE INFORMATION**			**Page Number**
Title:			
Identification	1a	Identify the report as a protocol of a systematic review	1
Update	1b	If the protocol is for an update of a previous systematic review, identify as such	
Registration	2	If registered, provide the name of the registry (such as PROSPERO) and registration number	PROSPERO 2021 CRD42021277073
Authors:			
Contact	3a	Provide name, institutional affiliation, e-mail address of all protocol authors; provide physical mailing address of corresponding author	1
Contributions	3b	Describe contributions of protocol authors and identify the guarantor of the review	
Amendments	4	If the protocol represents an amendment of a previously completed or published protocol, identify as such and list changes; otherwise, state plan for documenting important protocol amendments	11
Support:			
Sources	5a	Indicate sources of financial or other support for the review	
Sponsor	5b	Provide name for the review funder and/or sponsor	12
Role of sponsor or funder	5c	Describe roles of funder(s), sponsor(s), and/or institution(s), if any, in developing the protocol	
**INTRODUCTION**			
Rationale	6	Describe the rationale for the review in the context of what is already known	5
Objectives	7	Provide an explicit statement of the question(s) the review will address with reference to participants, interventions, comparators, and outcomes (PICO)	5
**METHODS**			

For peer review only

Eligibility criteria	8	Specify the study characteristics (such as PICO, study design, setting, time frame) and report characteristics (such as years considered, language, publication status) to be used as criteria for eligibility for the review	5
Information sources	9	Describe all intended information sources (such as electronic databases, contact with study authors, trial registers or other grey literature sources) with planned dates of coverage	7
Search strategy	10	Present draft of search strategy to be used for at least one electronic database, including planned limits, such that it could be repeated	7
Study records:			
Data management	11a	Describe the mechanism(s) that will be used to manage records and data throughout the review	
Selection process	11b	State the process that will be used for selecting studies (such as two independent reviewers) through each phase of the review (that is, screening, eligibility and inclusion in meta-analysis)	
Data collection process	11c	Describe planned method of extracting data from reports (such as piloting forms, done independently, in duplicate), any processes for obtaining and confirming data from investigators	
Data items	12	List and define all variables for which data will be sought (such as PICO items, funding sources), any pre-planned data assumptions and simplifications	7-8
Outcomes and prioritization	13	List and define all outcomes for which data will be sought, including prioritization of main and additional outcomes, with rationale	6-7
Risk of bias in individual studies	14	Describe anticipated methods for assessing risk of bias of individual studies, including whether this will be done at the outcome or study level, or both; state how this information will be used in data synthesis	9
Data synthesis	15a	Describe criteria under which study data will be quantitatively synthesised	
	15b	If data are appropriate for quantitative synthesis, describe planned summary measures, methods of handling data and methods of combining data from studies, including any planned exploration of consistency (such as I^2, Kendall's τ)	9
	15c	Describe any proposed additional analyses (such as sensitivity or subgroup analyses, meta-regression)	
	15d	If quantitative synthesis is not appropriate, describe the type of summary planned	
Meta-bias(es)	16	Specify any planned assessment of meta-bias(es) (such as publication bias across studies, selective reporting within studies)	10
Confidence in cumulative evidence	17	Describe how the strength of the body of evidence will be assessed (such as GRADE)	11, 14-15

For peer review only
